# Protease activities of vaginal *Porphyromonas* species disrupt coagulation and extracellular matrix in the cervicovaginal niche

**DOI:** 10.1038/s41522-022-00270-7

**Published:** 2022-02-21

**Authors:** Karen V. Lithgow, Vienna C. H. Buchholz, Emily Ku, Shaelen Konschuh, Ana D’Aubeterre, Laura K. Sycuro

**Affiliations:** 1 Department of Microbiology, Immunology and Infectious Diseases, University of Calgary, Calgary, AB Canada; 2 Calvin, Phoebe and Joan Snyder Institute for Chronic Diseases, University of Calgary, Calgary, AB Canada; 3 Alberta Children’s Hospital Research Institute, University of Calgary, Calgary, AB Canada; 4 International Microbiome Centre, University of Calgary, Calgary, AB Canada; 5 Present Address: Faculty of Medicine & Dentistry, University of Alberta, Edmonton, AB Canada; 6 Present Address: Department of Biological Sciences, University of Alberta, Edmonton, AB Canada

**Keywords:** Microbiome, Pathogens, Clinical microbiology

## Abstract

*Porphyromonas asaccharolytica* and *Porphyromonas uenonis* are common inhabitants of the vaginal microbiome, but their presence has been linked to adverse health outcomes for women, including bacterial vaginosis and preterm birth. However, little is known about the pathogenesis mechanisms of these bacteria. The related oral opportunistic pathogen, *Porphyromonas gingivalis*, is comparatively well-studied and known to secrete numerous extracellular matrix-targeting proteases. Among these are the gingipain family of cysteine proteases that drive periodontal disease progression and hematogenic transmission to the placenta. In this study, we demonstrate that vaginal *Porphyromonas* species secrete broad-acting proteases capable of freely diffusing within the cervicovaginal niche. These proteases degrade collagens that are enriched within the cervix (type I) and chorioamniotic membranes (type IV), as well as fibrinogen, which inhibits clot formation. Bioinformatic queries confirmed the absence of gingipain orthologs and identified five serine, cysteine, and metalloprotease candidates in each species. Inhibition assays revealed that each species’ proteolytic activity can be partially attributed to a secreted metalloprotease with broad substrate specificity that is distantly related to the *P. gingivalis* endopeptidase PepO. This characterization of virulence activities in vaginal *Porphyromonas* species highlights their potential to alter the homeostasis of reproductive tissues and harm human pregnancy through clotting disruption, fetal membrane weakening, and premature cervical remodeling.

## Introduction

The vaginal microbiome of healthy reproductive-age women is typically characterized by low species diversity, with *Lactobacillus* dominating the vaginal and ectocervical niches of the lower genital tract^1,2^. A community shift towards high species diversity, with overgrowth of anaerobic bacteria, is associated with increased risk of bacterial vaginosis (BV)^2,3^, acquisition and transmission of sexually transmitted infections^4–6^, preterm birth^7–9^, and cervical cancer^10–12^. Intriguingly, some women harboring a diverse cervicovaginal microbiome are healthy and asymptomatic^2^, suggesting a need to untangle how specific species contribute to poor outcomes. Among BV-associated bacteria, Gram-negative anaerobic rods corresponding to *Prevotella* and black-pigmented *Porphyromonas* species are frequently detected in vaginal samples and significantly associated with the *Bacteroides* morphotype from Nugent scoring^13^. While *Prevotella* is an abundant, species-rich, and relatively well-studied vaginal clade, comparatively little is known about *Porphyromonas* species inhabiting the human vagina. No *Porphyromonas* species is currently thought to be specific to the human urogenital tract, but *P. asaccharolytica*, *P. uenonis, P. bennonis*, and *P. somerae* (in decreasing order of cervicovaginal microbiome citation frequency) exhibit a preference for these niches^3,14,15^. *P. asaccharolytica* and *P. uenonis* colonize the vagina in 15–50% of healthy women and although their prevalence and abundance increase with BV, they are typically considered low abundance taxa^13,16–20^. Recent studies show these species are predictors of spontaneous preterm labor^9,21^, pelvic inflammatory disease^22,23^, human papillomavirus (HPV) infections progressing to cervical neoplasia^24,25^, and uterine cancer^26,27^. Thus, an improved understanding of the functional capacity of vaginal *Porphyromonas* species is needed.

To date, the only *Porphyromonas* species that has been well-characterized is *P. gingivalis*, a low abundance species in the oral microbiome of both healthy patients and those with gingivitis^28,29^. As a keystone species driving oral (plaque) biofilm formation and periodontal disease progression^30,31^, *P. gingivalis* contributes to local tissue destruction directly and indirectly through the induction of inflammatory processes^32,33^. *P. gingivalis* can also disseminate via the bloodstream to distal infection sites such as the endocardium and joints^34,35^. During pregnancy, *P. gingivalis* has been isolated from the placenta and amniotic fluid of women who delivered preterm^36–38^, and in mouse infection models, *P. gingivalis* induces preterm labor via inflammatory activation of the chorioamniotic membranes^34,39,40^. Pathogenesis mechanisms contributing to these outcomes include a wide array of proteolytic activities carried out by numerous secreted proteases. Among these are the gingipain family of cysteine proteases that drive periodontal disease progression^41^, hematogenic transmission to the placenta^29,40,42,43^, and preterm labor induction in mice^40^. The gingipains degrade many extracellular matrix components, including collagen^44,45^, and amplify their effects by activating and upregulating host matrix metalloproteases (MMPs) that also degrade collagen^46,47^. Furthermore, gingipains can degrade immune factors including immunoglobulins^44,48^, complement components^49,50^, cytokines^51,52^, clotting factors^53,54^, and antimicrobial peptides^55^, giving rise to a favorable immune environment for *P. gingivalis* colonization.

Proteolytic activity has been previously detected in vaginal fluid from patients with BV^56–58^ and characterized in clinical isolates of BV-associated bacteria^59,60^. In fact, collagenase (gelatinase) and caseinase activity of *P. asaccharolytica* (formerly *Bacteroides asaccharolyticus*) was previously reported in screens of *Bacteroides* species detected in human infections^61^ and clinical isolates from reproductive tract infections^60^. However, further characterization of the enzymes responsible was not conducted, and proteolytic activity of *P. uenonis* has yet to be explored. Given their phylogenetic relatedness and epidemiological similarity to *P. gingivalis*—exhibiting high prevalence, low abundance, and association with the disease—we sought to determine whether vaginal *Porphyromonas* species possess the broad-acting proteolytic virulence activity of the periodontal pathogen. In this study, we show that *P. asaccharolytica* and *P. uenonis* are capable of degrading multiple extracellular matrix components in the female genital tract. Our study furthermore reveals differences between the species, suggesting preterm birth-associated *P. asaccharolytica* may secrete more enzymes that contribute to collagenase activity. Finally, we identify and functionally characterize a *P. asaccharolytica* virulence factor—a metalloprotease that is highly conserved in *P. uenonis* and distantly related to the PepO endopeptidase in *P. gingivalis* and other *Porphyromonas* species^62,63^. We demonstrate this protein exhibits broad-acting proteolytic capacity, which may make it a key microbial virulence factor in the pathogenesis of gynecological and reproductive health conditions.

## Results

### Vaginal *Porphyromonas* species degrade type I collagen, type IV collagen, and casein using secreted proteases

**Fig. 1:**
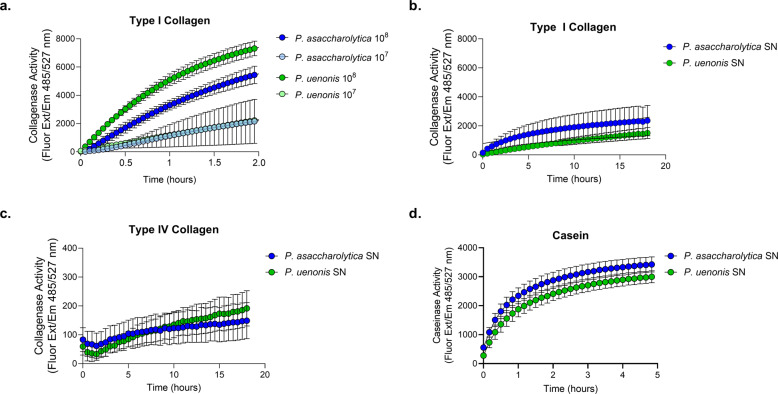
Proteolytic activity of vaginal *Porphyromonas* species. **a** Cell suspensions of *P. asaccharolytica* and *P. uenonis* at 10^7^ or 10^8^ CFU/reaction were incubated with fluorophore-conjugated type I collagen. Results are presented as mean ± standard error from three independent experiments performed in technical triplicate or quadruplicate. Collagen degradation was measured every 3 min by detecting the increase in fluorescence over a 2-h time course. **b**, **c** Cell-free supernatants of *P. asaccharolytica* and *P. uenonis* were incubated with fluorophore-conjugated **b** type I or **c** type IV collagen over an 18-h time course. Results are presented as mean ± standard error from seven independent experiments (type I collagen) and four independent experiments (type IV collagen) performed in technical triplicate. **d** Cell-free supernatants of *P. asaccharolytica* and *P. uenonis* were incubated with fluorescein (FITC)-conjugated casein over a 5-h time course. Results are presented as mean ± standard error from five independent experiments performed in technical triplicate. The mean fluorescence readings of the negative control were subtracted from experimental wells and relative fluorescence units (RFU) were plotted over time, with negative values adjusted to zero.

Collagenase activity of *P. asaccharolytica* and *P. uenonis* was evaluated using fluorescently quenched substrates (type I collagen or type IV collagen), where proteolytic digestion results in dequenching and measurable increases in fluorescence over time. Fluorometric collagenase assays confirmed that *P. asaccharolytica* and *P. uenonis* cell suspensions degrade type I collagen in a dose-dependent manner (Fig. 1a). Next, collagenase activity was measured in cell-free supernatants from *P. asaccharolytica* and *P. uenonis*, validating that both organisms secrete proteases capable of degrading type I and type IV collagen (Fig. 1b, c). Collagenase activity was further confirmed with gelatin zymography (Supplementary Fig. 2), where *P. asaccharolytica* supernatants produced three distinct high molecular weight zones of clearing (~85, 95, and 120 kDa), while *P. uenonis* supernatants generated four zones of clearing: three high molecular weight (~75, 90, and 110 kDa) and one low molecular weight (30 kDa). Due to its low-complexity tertiary structure, casein is regarded as a universal protease substrate that is highly susceptible to proteolytic degradation. Fluorometric assays revealed that supernatants from *P. asaccharolytica* and *P. uenonis* possess caseinase activity (Fig. 1d). This was further confirmed using agar-based casein degradation assays, where *P. asaccharolytica, P. uenonis*, and *P. gingivalis* cell suspensions all produced zones of clearing (Supplementary Fig. 3). Although *P. asaccharolytica* and *P. uenonis* can degrade similar substrates as *P. gingivalis*^41^, *P. gingivalis* showed substantially higher maximum fluorescence and area under the curve for collagen degradation (Supplementary Fig. 4 and Supplementary Tables 1, 2). The area under the curve for casein degradation by *P. gingivalis* was also much higher than for *P. asaccharolytica* or *P. uenonis*, while the maximum fluorescence and time to maximum fluorescence for casein degradation were comparable between all three *Porphyromonas* species (Supplementary Fig. 4 and Supplementary Table 2). To understand if proteolytic activity might contribute to pathogenesis in the female genital tract, we evaluated whether a common commensal vaginal microbe is also capable of degrading collagen and casein. No collagenase or caseinase activity was detected from *Lactobacillus crispatus* cell suspensions in the fluorometric collagenase or caseinase assays (Supplementary Fig. 5).

### *P. asaccharolytica* and *P. uenonis* inhibit fibrin clot formation through fibrinogen degradation

**Fig. 2:**
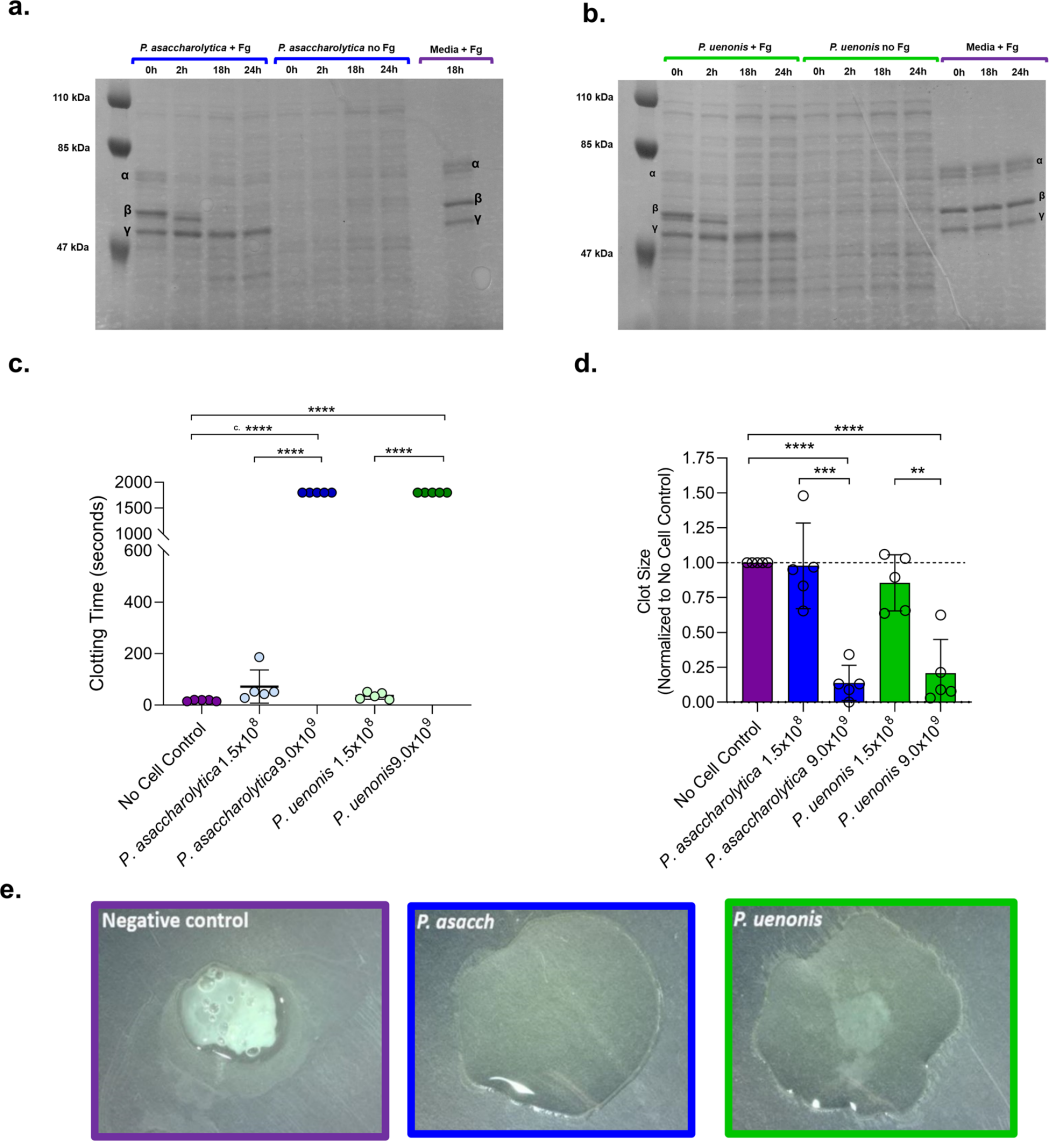
Vaginal *Porphyromonas* species degrade fibrinogen and impair clot formation. **a**, **b** SDS-PAGE of **a** *P. asaccharolytica* and **b** *P. uenonis* incubated with human fibrinogen (+Fg), saline (no Fg), or a cell-free media control (sBHI Media + Fg). Samples were assessed for fibrinogen degradation, indicated by the absence of bands corresponding to α, β, and γ fibrinogen chains in “*Porphyromonas* cells + Fg” treatments compared to “no Fg” or media controls. **c** Time from thrombin addition to fibrin clot formation. Citrated plasma was preincubated with *P. asaccharolytica, P. uenonis*, or no cell controls. Experiments were performed in technical duplicate and results are presented as mean ± standard error from five independent experiments. **d** Quantitative evaluation of clot size via well scan optical density measurements at the experimental endpoint. Experiments were performed in technical duplicate and control reactions without thrombin were used to blank the experimental reactions. Data were normalized to the average clot size of the no cell control within each experiment. Results are presented as mean ± standard error from five independent experiments. Significance was assessed by one-way ANOVA with Holm–Sidak’s multiple comparisons test (****p < 0.0001, ***p < 0.0003, **p = 0.003). **e** Endpoint qualitative evaluation of fibrin clots (>30 min) after clotting time assay with *P. asaccharolytica* (2.4 × 10^9^ CFU/reaction), *P. uenonis* (3.0 × 10^9^ CFU/reaction), or no cell control.

Since gingipains are known to degrade fibrinogen and exacerbate gum bleeding^41^, we next investigated whether *P. asaccharolytica* and *P. uenonis* proteases can degrade fibrinogen and impair fibrin clot formation. To evaluate direct fibrinogen degradation, cell-free media controls and cell suspensions of *P. asaccharolytica* or *P. uenonis* were incubated in the presence and absence of human fibrinogen over a 24-h time course. Samples removed at defined intervals were separated by SDS-PAGE and stained with Coomassie Brilliant Blue. When *P. asaccharolytica* or *P. uenonis* were incubated with fibrinogen, complete degradation of the fibrinogen α and β chains was observed after 18 h, while the γ chain remained intact (Fig. 2a, b). To determine whether fibrinogen degradation translates to impaired fibrin clotting, thrombin-induced fibrin clot formation was measured after *Porphyromonas* cell suspensions were preincubated with citrated plasma. A significant delay in clot formation was observed with the highest dose of *P. asaccharolytica* or *P. uenonis* (9.0 × 10^9^ CFU/reaction) compared to the no cell control (Fig. 2c, ****p < 0.0001) or a lower dose of *P. asaccharolytica* or *P. uenonis* (Fig. 2c; ****p < 0.0001). Turbidimetry at the experimental endpoint (30 min) allowed for quantitative evaluation of final clot size. In keeping with clotting times, fibrin clot size was significantly reduced in samples exposed to *P. asaccharolytica* or *P. uenonis* at 9.0 × 10^9^ CFU/reaction compared with the no cell control (Fig. 2d ****p < 0.0001) or lower doses of bacteria (Fig. 2d, ***p = 0.0003 for *P. asaccharolytica* and **p = 0.003 for *P. uenonis*). These findings were further confirmed by visual assessment of clot formation at assay endpoints (Fig. 2e).

### Identification of collagenase candidates in *P. asaccharolytica* and *P. uenonis*

Since our analyses confirmed previous reports that *P. asaccharolytica* and *P. uenonis* do not encode gingipain orthologs (Supplementary Note, Supplementary Fig. 1, and Supplementary Tables 3–5), we sought to identify other candidate proteins that may be responsible for the collagenolytic activity of vaginal *Porphyromonas* species. To identify putative collagenases amongst the 59 and 64 peptidases predicted for *P. asaccharolytica* and *P. uenonis* by the MEROPs database^64^, we cross-referenced a list of known and predicted microbial collagenases in the BRENDA enzyme information database^65^. This approach shortened the candidate peptidase list to 18 enzymes in *P. asaccharolytica* and 14 in *P. uenonis*. InterPro scans of each putative collagenase revealed proteins likely to be involved in cell wall synthesis or export machinery and narrowed the list to ten peptidases in *P. asaccharolytica* and nine peptidases in *P. uenonis* (Supplementary Fig. 1). Factoring in similarity to characterized collagenases, additional domains identified in InterPro, and the presence of secretion signals, a final short-list of the seven most promising candidate peptidases in each organism was generated (Tables 1, 2 and Supplementary Table 6). Intriguingly, each organism’s candidate collagenases could be organized into four groups: Ig-containing serine proteases (2x), C10 cysteine proteases (2x), M13 metalloproteases (1x), and U32 proteases (2x) (Tables 1, 2). Multiple sequence alignments of candidate collagenase pairs for a given type within each strain revealed low amino acid sequence identity (29–45%, Supplementary Table 7). Conversely, BLASTP searches between species allowed for the identification of orthologous protein pairs in *P. asaccharolytica* and *P. uenonis*, with amino acid identity ranging from 56 up to 97% (Supplementary Table 7). Notably, all within-genome candidate collagenases pairs had lower amino acid identity compared to the ortholog identified in the other *Porphyromonas* genome (Supplementary Table 7).

**Table 1:** Candidate collagenases in *P. asaccharolytica*.

Gene	Protease type	Collagenase IDs^e^	Secretion
Poras_1747	Ig-containing serine protease^a^	IPR013783 IPR026444 TIGR04183	Type IX^f^ Sec/SPII^g^
Poras_0168	Ig-containing serine protease^a^	IPR013783 IPR026444 TIGR04183	Type IX^f^
Poras_0079	M13 metalloprotease^b^	IPR024079	Sec/SPII^g^
Poras_1659	C10 protease^c^	IPR026444	Type IX^f^, Sec/SPI^g^
Poras_0891	C10 protease^c^	IPR026444	Type IX^f^ Sec/SPI^g^
Poras_0217	U32 collagenase^d^	U32.003 PF12392 PF01136 IPR020988 IPR001539 PS01276	N/A
Poras_0873	U32 collagenase^d^	PF01136 U32.003 IPR001539 PS01276	N/A

All peptidases from *P. asaccharolytica* DSM 20707 were populated in UniProt using the advanced search tool to identify proteins with MEROPs annotations. Peptidases were cross-referenced against protein annotation identifiers in known and predicted microbial collagenases (BRENDA enzyme number: EC3.4.24.3) and the most promising candidate collagenases were selected by exploration of each entry in InterPro, Uniprot, and MEROPS to identify important domains, secretion signals, and predicted inhibitors. Any proteins involved in cell wall synthesis were eliminated.

^a-d^Predicted inhibitors (MEROPS): ^a^Aprotinin, Bowman–Birk, Serpins, ^b^1,10-phenanthroline, phosphoramidon, ^c^Iodoacetamide, N-ethylmaleimide, ^d^N/A

^e^Collagenase IDs (BRENDA enzyme #: EC3.4.24.3, InterPro, Pfam or MEROPs IDs): IPR013783: Ig-like fold; IPR026444: C-term sec signal; TIGR04183: PorC sec signal; IPR024079: metallopeptidase catalytic domain; TIGR0483: Por sec system; PF01136; PF12392; U32.003: salmonella type collagenase; PF12392: Collagenase; PF01136: Peptidase U32; IPR020988: Peptidase U32, collagenase; IPR001539: Peptidase U23, bacterial collagenases; PS01276: Peptidase family U32; PF01136 Peptidase family U32; PF12392 collagenase.

^f^Type IX secretion determined by the presence of IDs: TIGR0483, IPR026444, or PF18962 from TIGR Fam, InterPro, and Pfam, in the protein C-terminus.

^g^SignalP prediction.

**Table 2:** Candidate collagenases in *P. uenonis*.

Gene^a^	Protease type	Collagenase IDs^f^	Secretion
Poru_01109	Ig-containing serine protease^b^	IPR013783 IPR026444 TIGR04183	Type IX^**g**^, Sec/SPI^**h**^
Poru_00939	Ig-containing Serine Protease^b^	IPR13783 IPR026444 TIGR0483	Type IX^**g**^, Sec/SPI^**h**^
Poru_00076	M13 Metalloprotease^c^	IPR024079	Sec/SPII^**h**^
Poru_01083	C10 Protease^d^	IPR026444 TIGR0483	Type IX^**g**^
Poru_00099	C10 Protease^d^	IPR026444 TIGR0483	Type IX^**g**^, Sec/SPI^**h**^
Poru_01540	U32 Collagenase^e^	PF01136 PF12392	N/A
Poru_00228	U32 Collagenase^e^	PF01136 PF12392	N/A

All peptidases from *P. uenonis* DSM 23387 were populated in UniProt using the advanced search tool to identify proteins with MEROPs annotations. Peptidases were cross-referenced against protein annotation identifiers in known and predicted microbial collagenases (BRENDA enzyme number: EC3.4.24.3) and the most promising candidate collagenases were selected by exploration of each entry in InterPro, Uniprot, and MEROPS to identify important domains, secretion signals, and predicted inhibitors. Any proteins involved in cell wall synthesis were eliminated.

^a^Locus tags correspond to L215DRAFT_XXXXX.

^b-e^Predicted inhibitors (MEROPS): ^b^Aprotinin, Bowman–Birk, Serpins, ^c^1,10-phenanthroline, phosphoramidon, ^d^Iodoacetamide, N-ethylmaleimide, ^e^N/A

^f^Collagenase IDs (BRENDA enzyme #: EC3.4.24.3, InterPro, Pfam, or MEROPs IDs): IPR013783: Ig-like fold; IPR026444: C-term sec signal; TIGR04183: PorC sec signal; IPR024079: metallopeptidase catalytic domain; TIGR0483: Por sec system; PF01136; PF12392; U32.003: salmonella type collagenase; PF12392: Collagenase; PF01136: Peptidase U32; IPR020988: Peptidase U32, collagenase; IPR001539: Peptidase U23, bacterial collagenases; PS01276: Peptidase family U32; PF01136 Peptidase family U32; PF12392 collagenase.

^g^Type IX secretion determined by the presence of IDs: TIGR0483, IPR026444, or PF18962 from TIGR Fam, InterPro, and Pfam, in the protein C-terminus.

^h^SignalP prediction.

Serine proteases within the short-list all contained Ig-like folds (Tables 1, 2), which are also present in the binding domain of the well-characterized collagen-degrading metalloproteases from *Clostridium histolyticum* (ColG, ColH)^66^. The candidate cysteine proteases contained type 9 secretion system signal domains and a SpeB domain (Tables 1, 2), indicating sequence similarity with *Streptococcus pyogenes* streptopain, a cysteine protease capable of cleaving host components such as fibrinogen, immunoglobulins, and complement proteins^67–69^. The candidate metalloproteases from *P. asaccharolytica* (Poras_0079) and *P. uenonis* (Poru_00076) each contained a catalytic collagenase domain in addition to an M13 type metallopeptidase domain and a predicted N-terminal secretion signal (Tables 1,2). Finally, two U32 collagenases were detected in each vaginal *Porphyromonas* species indicating an orthologous relationship with the *P. gingivalis* U32 collagenase PrtC^70,71^. Of note, none of these putative U32 collagenases were found to possess secretion signals indicative of localization to the extracellular space (Tables 1, 2).

### Vaginal *Porphyromonas* species encode metalloproteases targeting collagens, casein, and fibrinogen

**Fig. 3:**
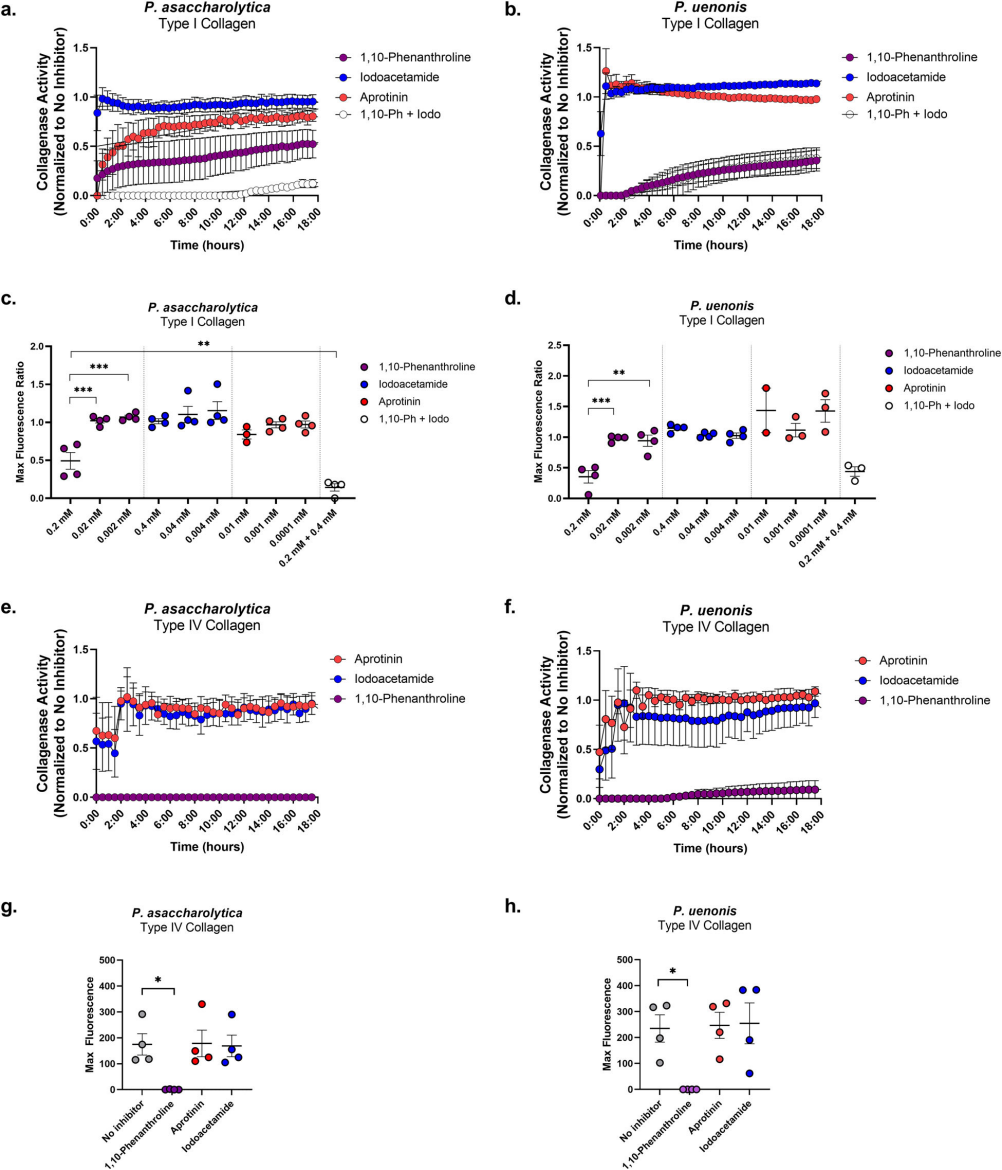
Vaginal *Porphyromonas* protease types display type I and type IV collagenase activity. **a**, **b** Type I collagen degradation of **a** *P. asaccharolytica* and **b** *P. uenonis* cell-free supernatants in the presence of 0.2 mM 1,10-phenanthroline, 0.4 mM iodoacetamide, 0.01 mM aprotinin or 0.2 mM 1,10-phenanthroline + 0.4 mM iodoacetamide. Results are normalized to the no inhibitor control (set to 1) and presented as mean ± standard error from four independent experiments. **c**, **d** Maximum fluorescence of **c** *P. asaccharolytica* and **d** *P. uenonis* secreted collagenase activity, normalized to the no inhibitor control and presented as mean ± standard error from four independent experiments, three independent experiments (*P. asaccharolytica* +0.01 mM aprotinin) or two independent experiments (*P. uenonis* +0.01 mM aprotinin) performed in technical triplicate. Statistical significance was assessed by one-way ANOVA and Tukey’s post hoc comparison *P. asaccharolytica*: 1,10-phenanthroline 0.2 vs. 0.02 mM ***p = 0.0009; 1,10-phenanthroline 0.2 vs. 0.002 mM ***p = 0.0005; 1,10-phenanthroline 0.2 vs. 0.2 mM 1,10-phenanthroline +0.4 mM iodoacetamide **p = 0.0092. *P. uenonis*: 1,10-phenanthroline 0.2 vs. 0.02 mM ***p = 0.0008; 1,10-phenanthroline 0.2 vs. 0.002 mM **p = 0.0012. **e**, **f** Type IV collagen degradation by **e** *P. asaccharolytica* and **f** *P. uenonis* cell-free supernatants in the presence of 0.2 mM 1,10-phenanthroline, 0.4 mM iodoacetamide, 0.01 mM aprotinin, or 0.2 mM 1,10-phenanthroline + 0.4 mM iodoacetamide. Results are normalized to the no inhibitor control and presented as mean ± standard error from four independent experiments. **g**, **h** Maximum fluorescence of **g** *P. asaccharolytica* and **h** *P. uenonis* secreted collagenase activity in the presence of inhibitors. Results are presented as mean ± standard error from four independent experiments performed in technical triplicate. Statistical significance was assessed by one-way ANOVA and Tukey’s post hoc comparison *P. asaccharolytica*: 1,10-phenanthroline 0.2 mM vs. no inhibitor *p = 0.0352.; *P. uenonis*: 1,10-phenanthroline 0.2 mM vs. no inhibitor *p = 0.0398. The mean fluorescence readings of the negative control were subtracted from experimental wells and relative fluorescence units (RFU) was plotted over time, with negative values adjusted to zero.

To further narrow down candidate enzymes responsible for collagenolytic and fibrinogenolytic activities, inhibitors of the predicted serine, cysteine, and metalloproteases (Tables 1, 2) were incorporated into functional assays. Cell-free supernatants from *P. asaccharolytica* and *P. uenonis* were incubated with type I collagen in the presence of three doses of 1,10-phenanthroline, iodoacetamide, or aprotinin to inhibit metallo-, cysteine, and serine proteases, respectively (Fig. 3a–d and Supplementary Fig. 6). Treatment of *P. asaccharolytica* supernatants with 1,10-phenanthroline resulted in decreased collagenase activity, with a statistically significant reduction in max enzyme activity observed with the highest dose of inhibitor (Fig. 3a, c; 0.2 vs. 0.02 mM ***p = 0.0009; 0.2 vs. 0.002 mM ***p = 0.0005, and Supplementary Fig. 6). Iodoacetamide treatment revealed a trend toward a dose-dependent decrease in collagenase activity in individual experiments (Supplementary Fig. 7), but this trend was not observed when multiple experiments were combined (Fig. 3a, c and Supplementary Fig. 6). However, when *P. asaccharolytica* supernatants were treated with a combination of 1,10-phenanthroline and iodoacetamide, there was a further reduction in collagenase activity compared to 1,10-phenanthroline alone (Fig. 3a, c, **p = 0.0092). There was no statistically significant decrease in the max collagenase activity secreted by *P. asaccharolytica* with aprotinin (Fig. 3a, c and Supplementary Fig. 6). For *P. uenonis*, 1,10-phenanthroline also inhibited collagenase activity (Fig. 3b, d and Supplementary Fig. 6), with a significant reduction in max collagenase activity (Fig. 3d; 0.2 vs. 0.02 mM ***p = 0.0008; 0.2 vs. 0.002 mM **p = 0.0012). However, treatment with iodoacetamide or aprotinin did not reduce *P. uenonis* collagenase activity (Fig. 3b, d and Supplementary Fig. 6) and the combination treatment of 1,10-phenanthroline and iodoacetamide did not provide an additional reduction in collagenase activity or max fluorescence (Fig. 3b, d) as observed in *P. asaccharolytica* (Fig. 3a, c).

**Fig. 4:**
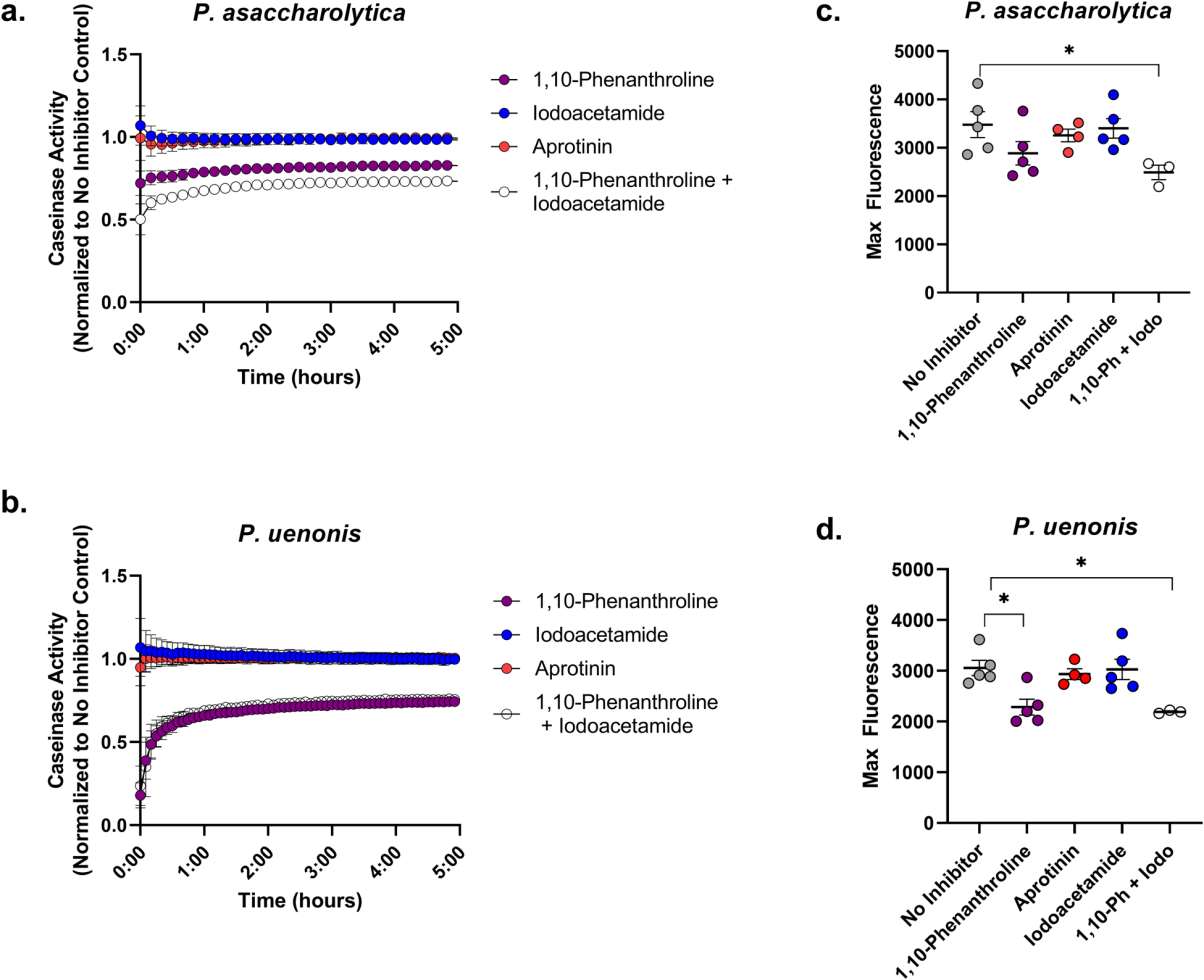
*P. asaccharolytica* and *P. uenonis* secreted metalloproteases have broad substrate specificity. **a**, **b** Cell-free supernatants of **a** *P. asaccharolytica* and **b** *P. uenonis* were incubated with fluorophore-conjugated casein in the presence of the metalloprotease inhibitor 1,10-phenanthroline (0.2 mM), the cysteine protease inhibitor iodoacetamide (0.4 mM), or the serine protease inhibitor aprotinin (0.01 mM). Casein degradation was measured every 10 min by detecting the increase in fluorescence over a 5-h time course. Results are presented as a ratio normalized to the no inhibitor control and presented as mean ± standard error from five independent experiments performed in technical triplicate. The mean fluorescence readings of the negative control were subtracted from experimental wells and relative fluorescence units (RFU) was plotted over time, with negative values adjusted to zero. **c** Maximum fluorescence of *P. asaccharolytica* supernatant caseinase activity in the presence of inhibitors. Results are presented as mean ± standard error from five independent experiments performed in technical triplicate. Statistical significance was assessed with a one-way ANOVA and Tukey’s post hoc comparison, where inhibitor combination vs. no inhibitor *p = 0.0413. **d** Maximum fluorescence of *P. uenonis* supernatant caseinase activity in the presence of inhibitors. Results are presented as mean ± standard error from five independent experiments performed in technical triplicate. Statistical significance was assessed with a one-way ANOVA and Tukey’s post hoc comparison, where 1,10-phenanthroline 0.2 mM vs. no inhibitor *p* = 0.0145, inhibitor combination vs. no inhibitor *p* = 0.0180.

Next, we evaluated whether the same protease classes degrade type IV collagen and casein. For both *Porphyromonas* species, the metalloprotease inhibitor 1,10-phenanthroline completely abrogated type IV collagenase activity from supernatants, while serine and cysteine protease inhibitors did not significantly reduce activity (Fig. 3e–h). *P. asaccharolytica* caseinase activity was similar to its type I collagenase activity (Fig. 4a, b) as proteolytic activity was reduced by treatment with 1,10-phenanthroline, and treatment with both 1,10-phenanthroline and iodoacetamide produced an additional downward shift in enzyme activity that resulted in a significant reduction in max fluorescence when compared to the no inhibitor control (Fig. 4a, c; *p* = 0.0413). In keeping with the type I collagen and type IV collagen degradation results, *P. uenonis* caseinase activity was significantly decreased by treatment with the metalloprotease inhibitor 1,10-phenanthroline (Fig. 4c, d; *p* = 0.018), while aprotinin and iodoacetamide treatment did not inhibit activity (Fig. 4b, d). Further to this, the 1,10-phenanthroline/iodoacetamide combination did not offer any additional reduction in protease activity (Fig. 4b, d).

**Fig. 5:**
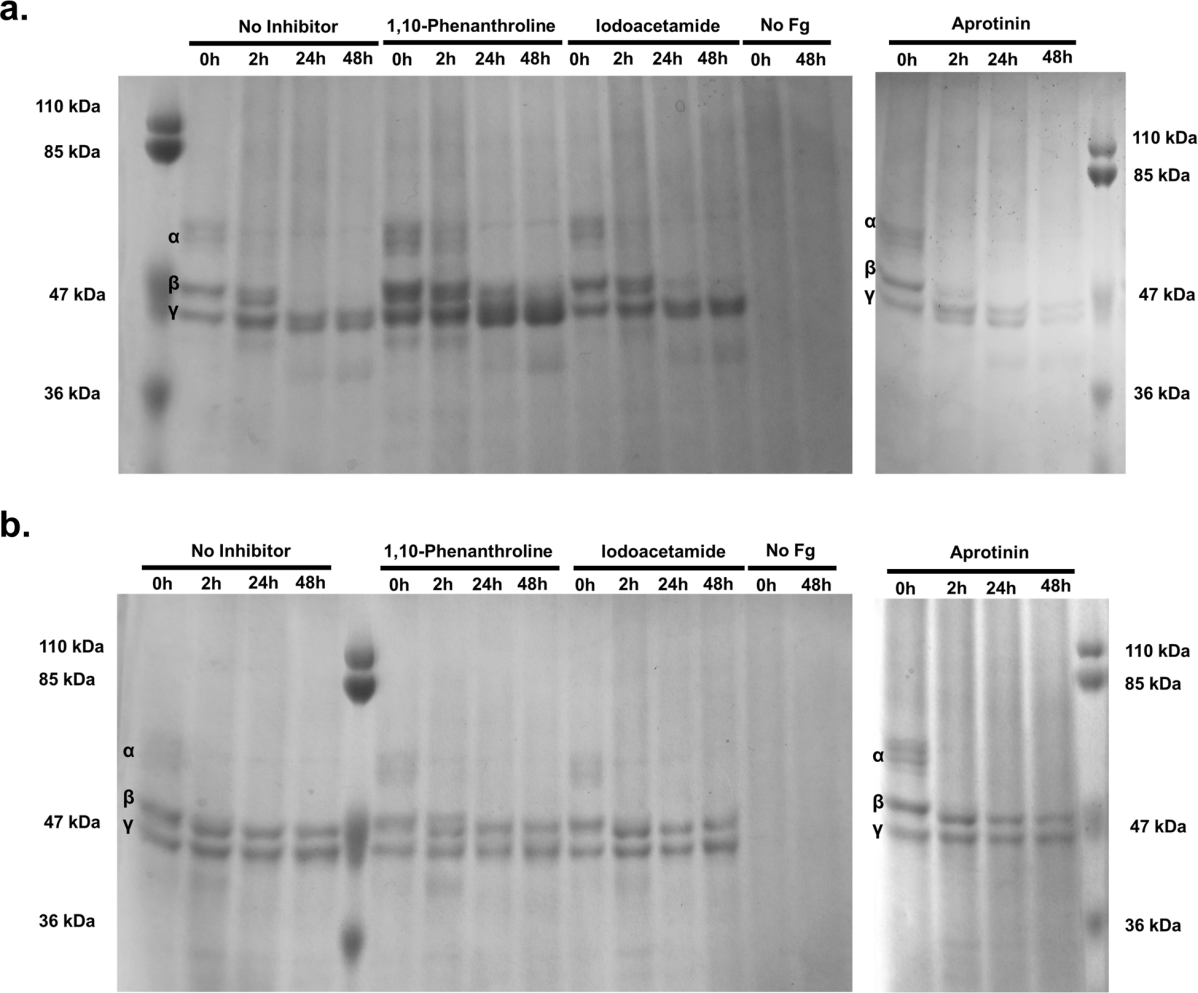
*P. asaccharolytica* metalloproteases degrade fibrinogen. **a** SDS-PAGE of *P. asaccharolytica* supernatants incubated with human fibrinogen in the presence of 1,10-phenanthroline (0.5 mM), iodoacetamide (1 mM), or aprotinin (0.01 mM) compared to no inhibitor and no fibrinogen controls. Samples collected over 48 h were assessed for fibrinogen degradation, indicated by the absence of bands corresponding to α, β, and γ fibrinogen chains as denoted within gel images. **b** SDS-PAGE of *P. uenonis* supernatants incubated with human fibrinogen in the presence of 1,10-phenanthroline (0.5 mM), iodoacetamide (1 mM), or aprotinin (0.01 mM) compared to no inhibitor and no fibrinogen controls. Samples collected over 48 h were assessed for fibrinogen degradation, indicated by the absence of bands corresponding to α, β, and γ fibrinogen chains.

Inhibitors were also incorporated into the fibrinogen degradation assay to determine whether fibrinogen is proteolyzed by the same enzyme classes as those observed in our collagen and casein experiments. Delayed fibrinogen degradation by *P. asaccharolytica* was observed in the presence of 1,10-phenanthroline, but not with aprotinin or iodoacetamide (Fig. 5a), suggesting that secreted metalloproteases from *P. asaccharolytica* contribute to fibrinogen degradation. Fibrinogen degradation patterns from *P. asaccharolytica* and *P. uenonis* supernatants (Fig. 5) differed from the results obtained with cell suspensions (Fig. 2a–b). For both species, the α chain was only partially degraded by the supernatants (Fig. 5). For *P. uenonis*, the β and γ chains remained intact for the 48-hour time course (Fig. 5b). Furthermore, *P. uenonis* partial degradation of the α chain was not impacted by the inhibitors included in this study (Fig. 5b).

**Fig. 6:**
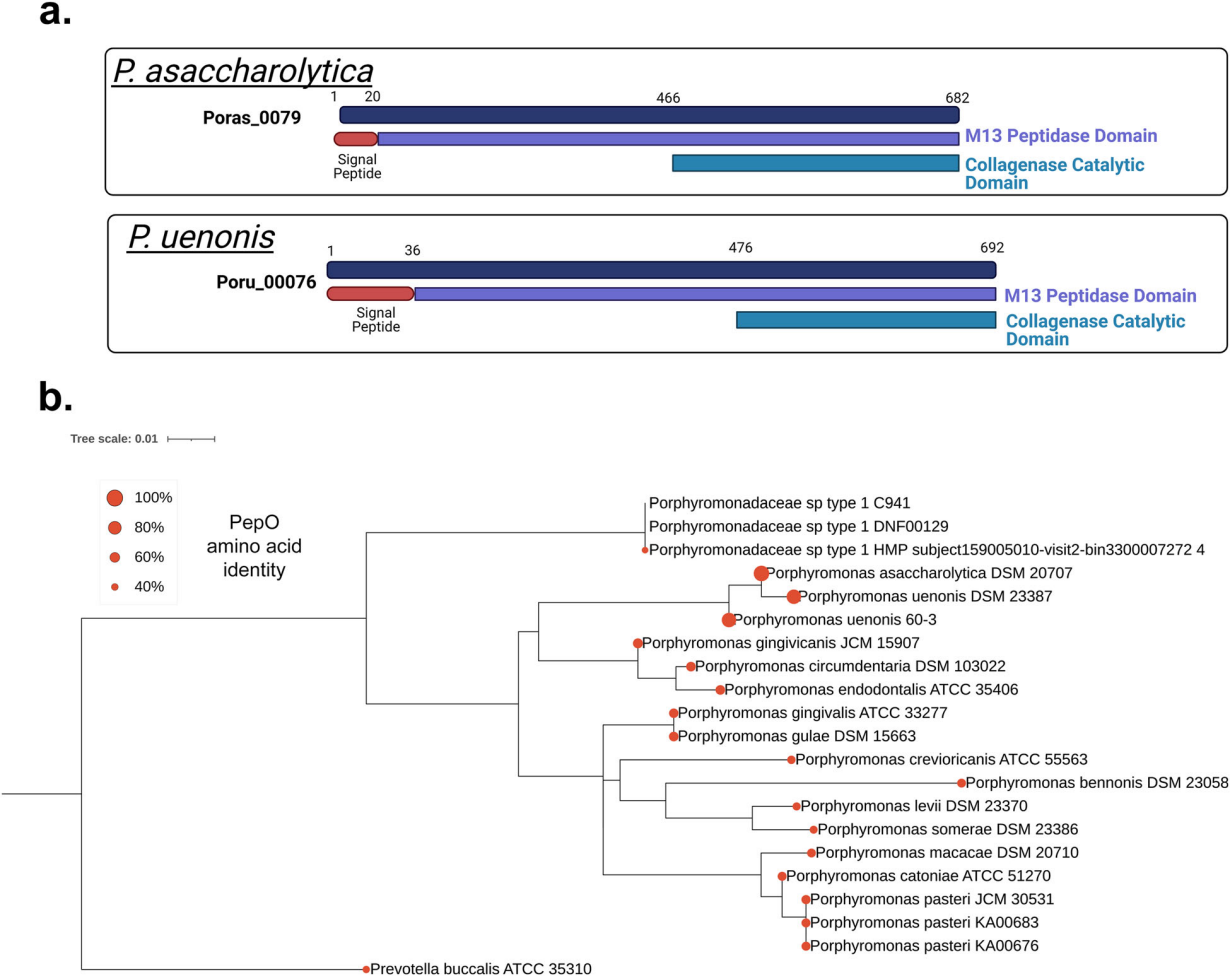
Secreted PepO metallopeptidases identified in *P. asaccharolytica* and *P. uenonis*. **a** Domain structure of candidate host-degrading PepO metalloproteases from *P. asaccharolytica* and *P. uenonis*. Created with BioRender.com. **b** 16S rRNA gene phylogeny of *Porphyromonas* species identified in the urogenital tract presented as a Maximum Likelihood tree rooted to *Prevotella buccalis* (same order, different family). Red circles on each leaf indicate the percent amino acid identity of that species’ PepO ortholog compared to *P. asaccharolytica* PepO (Poras_0079 = 100%). The taxon previously identified as uncultivated *Porphyromonas* species type 1 was found to encompass two cultured, but unsequenced isolates (DNF00129 and C941, >99% 16S rRNA gene identity over 1120 nt). We identified a metagenome-assembled genome (MAG) in IMG/MER that encoded a 958 bp 16S rRNA gene fragment >99.5% identical to those from the cultured isolates DNF00129 and C941. Since this MAG represents the only genome sequence available for this species, we used it in our PepO queries, identifying an ortholog 42% identical to *P. asaccharolytica* PepO. As this taxon’s PepO ortholog was more distantly related to *P. asaccharolytica* PepO than the *Prevotella buccalis* PepO ortholog (47% identical), *Porphyromonas* species type 1 may represent a novel genus or family; we, therefore, labeled this taxon *Porphyromonadaceae* sp. type 1. Our phylogenetic analysis, along with inquiries through the Genome Taxonomy Database furthermore indicated that vaginal isolates KA00683 and KA00676 should be designated as belonging to the species *P. pasteri*, with each containing a PepO ortholog 58–59% identical to that from *P. asaccharolytica*.

**Fig. 7:**
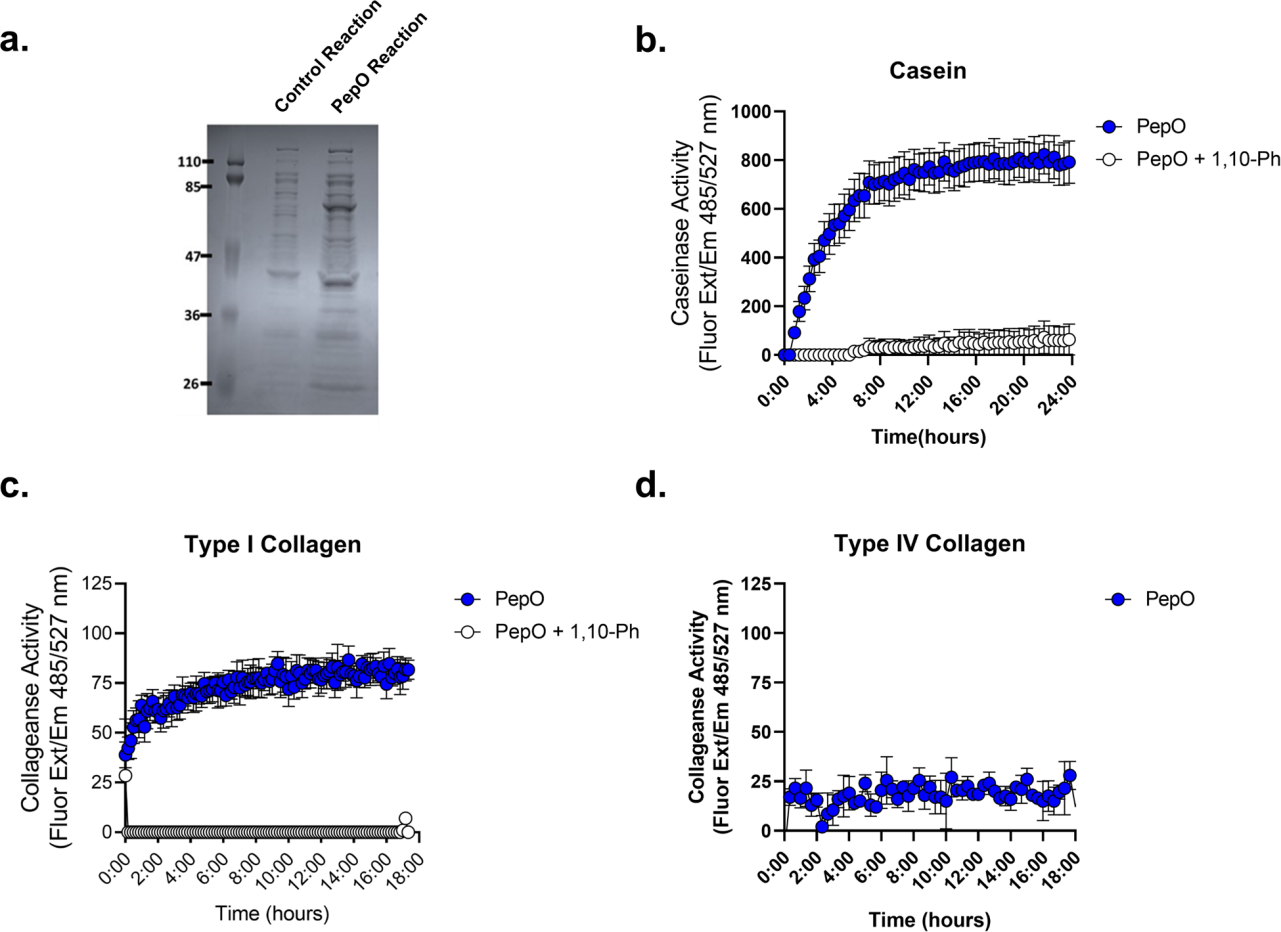
The *P. asaccharolytica* metalloprotease PepO degrades casein and type I collagen. **a** SDS-PAGE of control in vitro transcription/translation (myTXTL^®^) reaction (RNA polymerase only) and PepO myTXTL^®^ reaction. **b**–**d** PepO myTXTL^®^ reactions were incubated with **b** fluorescein (FITC)-casein or **c** fluorophore-conjugated type I collagen in the presence of 0.5 mM 1,10-phenanthroline. Casein degradation was measured by detecting the increase in fluorescence over a 24-h time course. Results are presented as mean ± standard error from three independent experiments. Collagen degradation was measured by detecting the increase in fluorescence over an 18-h time course. Results are presented as mean ± standard error from five independent experiments. **d** PepO myTXTL^®^ reactions were incubated with fluorophore-conjugated type IV collagen over an 18-h time course and results are presented as mean ± standard deviation from one independent experiment. The mean fluorescence readings of the negative control were subtracted from experimental wells and relative fluorescence units (RFU) was plotted over time, with negative values adjusted to zero.

Finally, we sought to further characterize the *Porphyromonas* M13 metalloproteases identified in our bioinformatics inquiries (Tables 1, 2 and Fig. 6a). Genomic exploration of other *Porphyromonas* species detected in the urogenital tract and commonly isolated from other human body sites revealed that the M13 metalloproteases are ubiquitous (Fig. 6b). Notably, the M13 metalloprotease in *P. gingivalis* has been previously characterized as PepO, a secreted endopeptidase involved in host attachment/invasion and proteolytic activation of endothelin, a potent peptide that induces vasoconstriction^62,63,72^. To determine whether the vaginal *Porphyromonas* M13 metalloproteases have collagenase and caseinase activity, Poras_0079 (*pepO)*, was cloned and expressed using the myTXTL^®^ in vitro transcription/translation system. Expression of PepO was confirmed via SDS-PAGE with the appearance of a 76 kDa protein in PepO reactions but not in the RNA polymerase only control reactions (Fig. 7a). Fluorescent protease assays revealed that *P. asaccharolytica* PepO is capable of degrading casein and type I collagen, but not type IV collagen (Fig. 7b–d). Further, the metalloprotease inhibitor 1,10-phenanthroline fully abrogated the type I collagenase and caseinase activities of PepO (Fig. 7b, c).

## Discussion

It is well established that mucinase activity is elevated during BV^73–76^ and attributed to *Gardnerella*^75,77^ and *Prevotella* species^75,78,79^. In support of this, sialidase activity has been utilized as a diagnostic marker for BV^78,80–83^. Although most studies have focused on the degradation of mucin glycans, proteolytic activity in the vaginal fluid has also been linked with BV status^56–58^ and described among predominant BV-associated bacteria^59,60^. Isolates of *Prevotella bivia* from women with preterm premature rupture of membranes (PPROM) were shown to secrete proteases that degrade elastin, collagen, casein, and gelatin^59^. In another study screening bacterial strains from women with PPROM, preterm labor, or puerperal infection, protease activity was confirmed in phylogenetically diverse Gram-negative and Gram-positive organisms. This included the BV-associated bacteria *Gardnerella vaginalis*, *Prevotella bivia* (formerly *Bacteroides bivius*), and *P. asaccharolytica* (formerly *Bacteroides asaccharolyticus*)^60^. The authors found that *P. bivia* and *P. asaccharolytica* exhibited gelatin and casein degradation, while *G. vaginalis* exclusively degraded casein. Our findings confirm the collagen/casein degradation capacity of *P. asaccharolytica* and extend these activities to *P. uenonis*. Furthermore, we demonstrate that both vaginal *Porphyromonas* species also degrade type IV collagen, commonly found in reproductive tissues^84,85^, and describe the diverse protease types capable of coordinating these activities.

Our in vitro analyses show that the proteolytic activities of *P. asaccharolytica* and *P. uenonis* are dose-dependent across a range of 10^7^–10^9^ cells/mL. With concentrations of these microbes reported at 10^3^–10^6^ cells/mL in healthy women^86,87^, and up to 10^9^ cells/mL in populations that include women with BV^15^, it is reasonable to expect that although vaginal *Porphyromonas* species are not high in relative abundance, they, like *P. gingivalis*, have the potential to cause disease in a protease- and community-dependent fashion^88^. To understand whether proteolytic activity is also observed among commensal bacteria that inhabit the vaginal niche, we investigated the proteolytic activity of *Lactobacillus crispatus*, demonstrating that *L. crispatus* is not capable of degrading type I collagen or casein. The absence of detectable proteolytic activity from a commensal *Lactobacillus* strain and the growing evidence for secreted proteolytic activity from BV-associated bacteria^59,60^, including vaginal *Porphyromonas* species, suggests that degradation of host proteins could be an important virulence trait of opportunistic pathogens in the female genital tract.

Our results demonstrate that vaginal *Porphyromonas* species are capable of directly degrading fibrinogen and impairing fibrin clot formation. Fibrinogen is detected in vaginal lavage fluid^89,90^ and is targeted by other reproductive pathogens^91,92^. Although the implications of altered fibrinogen levels in the female reproductive tract are not clear, impaired clotting functions could have severe consequences during labor and postpartum. Fibrinogen is known to increase substantially during pregnancy^93,94^, and decreased plasma fibrinogen levels have been associated with increased severity of postpartum hemorrhage^95^. Further to this, genetic fibrinogen abnormalities are significantly associated with miscarriage, placental abruption, and postpartum hemorrhage^96–99^.

Within the female genital tract, collagens are found within the vagina, cervix, uterus, and pelvic floor and their composition and content is significantly altered throughout pregnancy and during labor^100^. Vaginal and cervical tissue is primarily composed of fibrillar type I and III collagens, with type I collagen playing a critical role in tissue integrity^101–103^. Type IV collagen is typically found within basement membranes and is enriched within the placenta^84^. Both type I and type IV collagens are found within chorioamniotic membranes at the maternal–fetal interface^85^. During pregnancy, cervical collagens (type I, III) undergo a shift toward increased solubility and decreased abundance, contributing to cervical softening^104–106^, while increased collagenase activity is observed during cervical ripening to prepare for dilation and parturition^107,108^. Importantly, cervical remodeling during term and preterm labor occurs via the same mechanisms, with host matrix metalloproteinases (MMPs) coordinating cervical collagen degradation^109^. Premature preterm rupture of the membrane (PPROM) has been associated with infection^110,111^, increased host MMP collagenase activity^112,113^, and decreased collagen content^114^. Furthermore, microbial collagenases can reduce the tensile strength of chorioamniotic membranes ex vivo^115^, and collagenase activity has been detected in clinical isolates from PPROM patients^59,60^. Taken together, these findings imply that host and microbial modulation of collagen within the cervix and chorioamniotic membranes could play critical roles in preterm labor and PPROM. In the present study, our findings indicate that proteases secreted by *P. asaccharolytica* and *P. uenonis* are capable of degrading both type I and type IV collagens, uncovering a possible mechanism for how these microbes contribute to the initiation of preterm labor.

Although *P. asaccharolytica* and *P. uenonis* are phylogenetically related to the opportunistic periodontal pathogen *P. gingivalis* and our investigations demonstrated that *P. asaccharolytica* and *P. uenonis* possess gingipain-like activities, including degradation of type I and type IV collagen, casein, and fibrinogen, gingipain homologs are absent in *P. asaccharolytica* and *P. uenonis*. Our bioinformatics inquiries identified five candidate secreted collagenases in each vaginal *Porphyromonas* species. Interestingly, within-genome pairs of candidate collagenases had lower amino acid identity than interspecies pairs, suggesting that within-genome pairs are paralogs that resulted from a gene duplication event prior to speciation of *P. asaccharolytica* and *P. uenonis*^116^. By incorporating protease inhibitors into our functional assays, we determined that *Porphyromonas* secreted metalloproteases degrade collagens (type I, IV), casein, and fibrinogen. Although *P. asaccharolytica* and *P. uenonis* possess the same candidate collagenases, only *P. asaccharolytica* appears to secrete both metallo and cysteine proteases in the experimental conditions used in our study. Future investigations will need to address whether pH or redox state may affect the activity of the cysteine proteases. Additionally, due to the general proteolytic activity of the metallo and cysteine proteases, it is plausible that secreted proteases may degrade other proteins in the supernatants, including other proteases. Our results also indicate that *P. uenonis* may degrade fibrinogen using both secreted and cell surface-associated proteases, as *P. uenonis* cell suspensions degraded the α and β chains of fibrinogen, while cell-free supernatants partially degraded the α chain only. Interestingly, the secreted *P. uenonis* protease that contributes to partial degradation of the fibrinogen α chain is not impacted by the inhibitors included in this study, implying that the secreted fibrinogenolytic enzyme from *P. uenonis* is distinct from the secreted collagen and casein degrading enzymes.

Since 1,10-phenanthroline inhibited the secreted proteolytic activity of *P. asaccharolytica* and *P. uenonis*, the M13 metalloproteases (distant orthologs of *P. gingivalis* PepO [PgPepO]) were expected to confer the observed protease activity. Our results confirm that *P. asaccharolytica* PepO degrades type I collagen and casein, but not type IV collagen, suggesting there is an additional type IV collagen-degrading metalloprotease in *Porphyromonas* supernatants. Previous work characterizing *P. gingivalis* PepO revealed sequence conservation with the human endothelin converting enzyme 1 (ECE-1), which proteolytically processes inactive endothelin (big endothelin) into active endothelin. Activated endothelin peptides can induce vasoconstriction and cellular proliferation, alter vascular permeability and activate inflammatory cells^117,118^. PgPepO was confirmed to possess ECE-1 like activity, converting all three subtypes of big endothelin to active endothelin^62^. Numerous studies have also demonstrated that PgPepO plays a role in cellular invasion and intracellular survival of *P. gingivalis*^63,72^. However additional substrates for this endopeptidase and the functional consequences of bacterial endothelin activation have yet to be explored.

PepO orthologs have also been characterized in select *Lactobacillus* species: *Lactobacillus lactis* and *Lactobacillus rhamnosus* PepO can proteolyze casein, but these enzymes are either confirmed or predicted to localize to the cytoplasm^119,120^. PepO has also been explored in *Streptococcus* species, including *Streptococcus pneumoniae* and *Streptococcus pyogenes* (Group A Streptococci; GAS). In *S. pneumoniae*, PepO is detected on the bacterial cell surface and in culture supernatants. *S. pneumoniae* PepO binds to host cells and fibronectin, facilitating bacterial adhesion and invasion^121^. *S*. *pneumoniae* PepO also interacts with the host immune system, but there is conflicting evidence on whether this contributes to immune evasion or activation^121–124^. Surprisingly, none of these studies have shown any proteolytic activity of *S. pneumoniae* PepO. In GAS, PepO has been detected both in the cytoplasm and as a secreted protein^125,126^. Similar to *S. pneumoniae*, GAS PepO contributes to complement evasion^126^. GAS PepO also participates in regulating quorum sensing via direct degradation of peptide pheromones secreted by GAS^125^. Taken together, this body of work highlights the broad substrate specificity and diverse functionality of bacterial PepO. Our work characterizing *P. asaccharolytica* PepO demonstrates degradation of host extracellular matrix (type I collagen), adding another substrate to the proteolytic repertoire of PepO. The findings that PepO can degrade regulatory proteins secreted by host cells (*P. gingivalis* PepO: endothelin) and bacterial cells (GAS PepO: quorum sensing peptides) suggest that PepO enzymes could play a role in the dysregulation of proteolytic cascades. Further investigation is needed to better understand the substrates targeted by PepO proteins in complex mucosal body sites such as the female reproductive tract to reveal how this enzyme contributes to the pathogenesis of phylogenetically diverse bacteria.

## Methods

### Bacterial strains and growth conditions

*Porphyromonas asaccharolytica* CCUG 7834 (type strain, identical to DSM 20707, ATCC 25260, and JCM 6326), *Porphyromonas uenonis* CCUG 48615 (type strain, identical to DSM 23387, ATCC BAA-906, and JCM 13868), *P. gingivalis* W50 ATCC 53978 and *Lactobacillus crispatus* CCUG 42897 were cultured anaerobically on 1.5% brucella agar (BD Biosciences, Franklin Lakes, MD) supplemented with 5% defibrinated sheep’s blood (Dalynn Biologicals, Calgary, AB). For liquid cultivation, supplemented brain heart infusion (sBHI) was prepared by supplementing BHI (BD) with 2% gelatin (BD), 1% yeast extract (ThermoFisher Scientific, Burnaby, BC), 0.8% dextrose (BD) and 0.1% starch (ThermoFisher). Solid and liquid cultivation was conducted at 37 °C in an AS-580 anaerobic chamber (Anaerobe Systems, Morgan Hill, CA). Bacterial suspensions were prepared by harvesting cells from the solid medium after growth for 16–24 h (*P. gingivalis, L. crispatus*) or 36–48 h (*P. asaccharolytica* and *P. uenonis)* and resuspending cells in sBHI, sBHI (no gelatin), BHI (no supplements), or PBS. Optical density (600 nm, OD600) of bacterial suspensions was measured with a Genesys 300 visible spectrophotometer (ThermoFisher Burnaby, BC) and colony forming units per mL (CFU/mL) was calculated using empirically determined CFU/mL/OD600 for each strain. Serial dilution spot plating was used to verify the CFU/mL of the starting suspensions. Cell-free supernatants (SNs) were harvested during late-log to early stationary phase for each species (*P. asaccharolytica* OD600 1.3–1.8 [1.7 × 10^9^–2.3 × 10^9^ CFU/mL]; *P. uenonis* OD600 0.8–1.2 [2.1 × 10^9^–3.2 × 10^9^ CFU/mL]).

### Collagenase/caseinase assays

Cell suspensions or cell-free supernatants were tested for collagenase activity with the EnzChek Gelatinase/Collagenase assay kit (Invitrogen, Carlsbad, CA) using fluorophore-labeled DQ^TM^ gelatin conjugate (predominantly type I collagen, Invitrogen) or a fluorophore-labeled DQ^TM^ type IV collagen conjugate from the human placenta (Invitrogen). Cell-free supernatants were harvested from liquid cultures by centrifuging at 10,000 × *g* for 10 min at room temperature before the supernatant was filter-sterilized (0.2 µm; Pall Laboratory, Mississauga, ON) and stored at −20 °C. Reactions were prepared by mixing 20 µL of the substrate at 0.25 mg/mL with 80 µL of reaction buffer and 100 µL of bacterial suspension, cell-free supernatant, or media in black optical bottom 96-well plates (Greiner Bio-One, Monroe, NC). Reactions testing general protease activity used fluorescein (FITC)-labeled casein as a substrate and were prepared by mixing 20 µL of the substrate at 50 µg/mL with 80 µL of Tris-buffered saline and 100 µL of cell-free supernatant. Plates were incubated in a Synergy H1 microplate reader (BioTek, Winooski, VT) at 37 °C in atmospheric conditions. Kinetic fluorescence reads were measured after 7 s shaking every 3–30 min at 485 nm excitation/527 nm emission. The mean fluorescence readings of the negative control (substrate in sBHI media) were subtracted from experimental wells and relative fluorescence units (RFU) were plotted over time, with negative values adjusted to zero.

### Casein plate assay

Casein plates were prepared by autoclaving three solutions: 30 g/L instant skim milk power (Pacific Dairy), 19 g/L brain heart infusion (BHI, BD Biosciences), and 30 g/L agar (Fisher Scientific). The solutions were combined in equal volume and 10 mL was added to 100 × 15 mm petri dishes (Fisher Scientific) to solidify at room temperature. Bacterial suspensions were prepared in PBS and 5 µL was spotted onto casein agar plates. Zones of clearance were measured for each spot after incubating the plates at 37 °C under anaerobic conditions for 3 to 6 days.

### Zymography

The total protein content of *Porphyromonas* cell-free supernatants was determined using a bicinchoninic acid microplate assay (BCA; Pierce, Rockford, IL). Cell-free supernatants were diluted to 8 mg/mL and 5 µL of the sample was combined with 5 µL of Novex^TM^ Tris-Glycine SDS Sample Buffer (Invitrogen) to load 40 µg per well. Samples were separated on Novex^TM^ 10% Zymogram Plus (Gelatin; Invitrogen) protein gels at a constant voltage of 125 V in Novex^TM^ 1X Tris-Glycine Running Buffer (Invitrogen). After separation, gels were incubated in Novex^TM^ 1X Renaturing Buffer for 30 min at room temperature with gentle agitation, followed by two consecutive incubations in Novex^TM^ 1X Developing Buffer: room temperature for 30 min and 37 °C for 16 h. Gels were then stained in Coomassie brilliant blue R-250 solution (Fisher Scientific) and de-stained in 5% methanol/7.5% acetic acid in distilled water. The unprocessed scan of the zymogram gel is available in the Supplementary Information (Supplementary Fig. 8).

### Clotting assays

Bacterial strains were harvested from a solid medium and suspended in 13 mM sodium citrate. Suspensions were centrifuged at 10,000 × *g* for 7 min at room temperature and resuspended in 13 mM sodium citrate. Duplicate cell suspensions, or cell-free controls, were incubated with 50 µL of sterile-filtered bovine plasma (Quad Five, Ryegate, MT) for 30 min at 37 °C under anaerobic conditions. After incubation, samples were blinded and centrifuged at 10,000 × *g* for 7 min at room temperature, and 50 µL of HEMOCLOT thrombin time reagent (Hyphen Biomed, Neuville-sur-Oise, France) was added to each reaction. The clotting time for each blinded sample was estimated using a stereo microscope to directly visualize clot formation, indicated by the presence of white precipitate, tendril formation, or increased viscosity of the samples. The clotting time for samples that did not form visible clots was recorded as 1800 s. To further evaluate final clot size, samples were transferred to a clear 96-well plate and OD (405 nm) was measured for the entire well using the well area scan feature of the Synergy H1 microplate reader (BioTek).

### Fibrinogen degradation assay

*Porphyromonas* cell suspensions (10^7^ CFU/reaction), sBHI media controls, or *Porphyromonas* cell-free supernatants (240 µg of total protein) were incubated with 120 µg of human fibrinogen (Sigma-Aldrich, St. Louis, MO). Reaction mixtures were incubated at 37 °C under anaerobic conditions or in an atmosphere of 5% CO_2_ for cell suspension or cell-free supernatants, respectively. Samples from each time point (cell suspensions: 0, 2, 18, 24 h; supernatants: 0, 2, 24, 48 h) were collected, mixed 1:1 with Novex^TM^ 2X sample buffer (Invitrogen) with dithiothreitol (DTT, Fisher Scientific), heated at 95 °C for 10 min, and separated on Novex^TM^ 10% Tris-Glycine polyacrylamide pre-cast gels (Invitrogen) at a constant voltage of 180 V. Gels were stained in 0.25% Coomassie brilliant blue R-250 solution (Fisher Scientific) and de-stained in 5% methanol/7.5% acetic acid in distilled water. Fibrinogen degradation was evaluated qualitatively by visualization of fibrinogen α chain (63.5 kDa), β chain (56 kDa), and γ chain (47 kDa) between experimental and control samples over the time-course. Gels are derived from the same experiments and were processed in parallel. Unprocessed scans of fibrinogen degradation protein gels are available in the Supplementary Information (Supplementary Figs. 9, 10).

### Bioinformatic analyses

#### Gingipain ortholog queries

Gingipain amino acid sequences RgpA (PGN_1970; P28784), RgpB (PGN_1466; P95493), and Kgp (PGN_1728; B2RLK2) were obtained from *P. gingivalis* ATCC 33277 through UniProtKB. Each sequence was queried against all available *P. asaccharolytica* (DSM 20707; PR426713P-I) and *P. uenonis* (DSM 23387; 60-3) genomes using the “Selected Genomes” protein Basic Local Alignment Search Tool (BLAST; default settings) in the IMG/MER database to identify potential orthologs (Supplementary Fig. 1A). Each gingipain amino acid (AA) sequence was also submitted to the Pfam database and all Pfam IDs and names were recorded. Gingipain Pfam IDs were searched against all available *P. asaccharolytica* (DSM 20707; PR426713P-I) and *P. uenonis* (DSM 23387 [IMG Genome ID 2585427891 and 2528311143]; 60-3) strains using the advanced gene search function in the IMG/MER database (Supplementary Fig. 1A). All sequences returned by these IMG/MER BLAST and Pfam ID searches were used to query *P. gingivalis* ATCC 33277 in a reciprocal BLAST search with the National Center for Biotechnology Information (NCBI) protein BLAST tool (Supplementary Fig. 1A). Select hits were aligned with the *P. gingivalis* gingipains across their full length using the EMBL-EMI Clustal Omega alignment tool^127^.

#### Candidate collagenase queries

Peptidases from *P. asaccharolytica* CCUG 7834 and *P. uenonis* 60-3 were identified by searching for entries with MEROPS peptidase annotations in UniProt^64,128^. For the microbial peptidases from *P. asaccharolytica* and *P. uenonis*, the following enzyme information was exported from UniProtKB for each entry: gene ontology (biological process and molecular function), MEROPs, Pfam, PANTHER, PROSITE, SMART, SUPFAM. Next, a microbial collagenase enzyme number (EC3.4.24.3) was identified in BRENDA^65^ and searched against the UniProtKB database^128^, generating a list of 3417 entries corresponding to predicted and confirmed microbial collagenases. These microbial collagenase identifiers were cross-referenced against the exported peptidase information from *P. asaccharolytica* and *P. uenonis* to generate a short-list of 14–18 candidate collagenases in *P. asaccharolytica* and *P. uenonis* (Supplementary Fig. 1B). Short-list candidates were explored in UniProt and InterPro Scan^128,129^ to eliminate any proteins involved in cell wall synthesis or export machinery. Sequences were evaluated for the presence of secretion signals using SignalP v.5.0^130^ or InterPro^129^. Presence of the IDs: TIGR0483, IPR026444, or PF18962 from TIGR Fam, InterPro, and Pfam, respectively, within protein C-termini was indicative of secretion via the type IX secretion system. Since the MEROPs peptidase database only contained information for *P. uenonis* 60-3, IMG BLAST searches were used to identify the corresponding candidate collagenase in the experimental strain used in this study, *P. uenonis* CCUG 48615. Sequence identity and InterPro scans were evaluated for the top hit from each BLAST search in *P. uenonis* CCUG 48615 (Supplementary Table 6).

#### Phylogenetic analysis

16S rRNA gene sequences were collected using the Integrated Microbial Genomes & Microbiomes (IMG/MER)^131^ or National Center for Biotechnology Information (NCBI) databases for each *Porphyromonas* species reported in the human urogenital tract by Acuna-Amador in their comprehensive review^14^. Uncultured and recently cultured *Porphyromonas* species were also included^15,132^. Nomenclature choices were informed by the average nucleotide identity (ANI) tool within IMG/MER and the Genome Taxonomy Database (GTDB, Release 06-RS202, April 27, 2021)^133^. Whenever possible, type strain and near full-length sequences were selected. Multiple sequence alignment was performed with SINA aligner (v.1.2.11)^134^ using Silva’s Alignment, Classification and Tree Service (ACT)^135^. The phylogenetic tree was also computed through ACT using RAxML v.8.2.9^136^ with the Gamma model for likelihoods. The tree was edited using the interactive Tree of Life (iTOL^137^). The AA identity of PepO orthologs in each *Porphyromonas* species was queried through BLASTP searches in IMG/MER and NCBI using the *P. asaccharolytica* AA sequence as the query. With the exception of *P. uenonis* 60-3, the AA identity reported corresponds with a hit showing >98% query coverage.

#### Protease inhibition assays

The metalloprotease inhibitor, 1,10-Phenanthroline (Invitrogen), was prepared as a 2 M stock solution in ethanol. The cysteine protease inhibitor iodoacetamide (G-Biosciences, St. Louis, MO, or Sigma-Aldrich) was prepared as a 10 mM stock solution in HyPure H_2_O (Cytiva Life Sciences, Marlborough, MA). The serine protease inhibitor aprotinin (Roche, Mississauga, ON) was prepared as a 0.1 mM stock solution in HyPure H_2_O. Working solutions of protease inhibitors were diluted in EnzChek reaction buffer (Invitrogen) or TBS and incorporated into collagenase, caseinase, and fibrinogen degradation assays at the concentrations reported in the figure legends.

### Expression construct cloning and in vitro transcription/translation

The Poras_0079 (PepO; IMG Gene ID 2504823953) DNA fragment encoding amino acid residues C21 to W682 was PCR amplified from a *P. asaccharolytica* DSM 20707 genomic DNA extraction (Qiagen DNeasy Blood & Tissue Kit, Germantown, MD) using the forward (5′-ATATCCATGGCTTGTAACAAGAAGCAGGAGAATC-3′) and reverse primers (5′-ATATCCCGGGCCAGACCACGACACGCTC-3′). The amplicon was cloned into the pTXTL-T7p14-aH plasmid (replacing alpha-hemolysin, Daicel Arbor Biosciences, Ann-Arbor, MI) using NcoI-HF and SmaI (New England Biolabs, Ipswitch, MA). The new plasmid construct (pSLP15) was transformed into *Escherichia coli* DH5α chemically competent cells and prepped using the QIAprep spin miniprep kit (Qiagen). Plasmid concentrations were determined using the Qubit dsDNA broad-range assay kit with a Qubit 3 fluorometer (Invitrogen). In vitro myTXTL^®^ reactions were prepared by combining 5 nM of pSLP15, 1 nM of pTXTL-P70a-T7rnap (expressing the T7 RNA polymerase, Daicel Arbor Biosciences), and 9 µl of myTXTL^®^ Sigma 70 Master Mix (Daciel Arbor Biosciences) to a final volume of 12 µL with HyClone HyPure water H_2_O (Cytiva); negative controls included 1 nM pTXTL-P70a-T7rnap and myTXTL^®^ Sigma 70 Master Mix only. For each reaction, 10 µL was transferred to a PCR-clean polypropylene V bottom 96-well plate and covered with a silicone seal (Eppendorf, Mississauga, ON). Reactions were incubated at 29 °C for 16 h and final reactions stored at −20 °C. To assess proteolytic activity, control (RNAP only) and PepO reactions were incorporated into fluorescent collagenase and caseinase assays. For caseinase assays, myTXTL^®^ reactions were diluted 60-fold in TBS and 10 µL was added to each well. For type I and type IV collagenase assays, 10 µL of undiluted myTXTL^®^ reactions were added to each well. Caseinase and type I collagenase assays were also conducted in the presence of 0.5 mM 1,10-phenanthroline. Mean fluorescence readings of the negative control (substrate with RNAP myTXTL^®^ reaction) were subtracted from the experimental wells and relative fluorescence units (RFU) were plotted over time, with negative values adjusted to zero. The unprocessed scan of the myTXTL^®^ reaction protein gel is available in the Supplementary Information (Supplementary Fig. 11).

### Bioinformatics and statistical analyses

Details of our bioinformatics analyses are provided in the Methods. Statistics were performed in GraphPad Prism (GraphPad, San Diego, CA) or Stata 15 (StataCorp, College Station, TX) and graphs were prepared in GraphPad. GraphPad was used to assess data variance and normality (Shapiro–Wilk test) and statistical significance was evaluated as described in figure legends. All schematic illustrations were created with BioRender.com.

### Reporting Summary

Further information on research design is available in the Nature Research Reporting Summary linked to this article.

## Supplementary information

Supplemental Text & Figures

Reporting Summary

## Data Availability

The authors confirm that the data supporting the findings of this study are available within the paper and Supplementary files. Additional data are available from the corresponding author upon reasonable request. The unprocessed protein gel scans are available in the Supplemental Information (Supplemental Figs. 8–11). *P. gingivalis* ATCC 33277 gingipain protein sequences were accessed via UniProtKB using the following accession numbers: RgpA (PGN_1970; P28784), RgpB (PGN_1466; P95493), and Kgp (PGN_1728; B2RLK2). Gingipain BLASTP and Pfam searches were conducted against *P. asaccharolytica* and *P. uenonis* strains in IMG using the following IMG genome IDs: 649989985, 2504756018, 643886142, 2585427891, 2528311143. Candidate collagenases in *P. asaccharolytica* DSM 20707 and *P. uenonis* 60-3 were accessed via UniProtKB using the taxon identifiers 879243 and 596327, respectively. The protein sequence for the *P. asaccharolytica* PepO protease was retrieved through UniProtKB with the accession ID F4KL59. Cultured and uncultured vaginal *Porphyromonas* and *Prevotella* species’ 16S rRNA gene sequences were drawn from public databases as follows: Set I – (NCBI genome accession/locus tag): *P. bennonis* DSM 23058 (PRJNA169760/B088DRAFT_10000), *P. asaccharolytica* DSM 20707 (NC_015501/Poras_R0016), *P. uenonis* 60-3 (NZ_ACLR00000000/PORUE0001_1896), *P. endodontalis* ATCC 35406 (NZ_ACNN00000000/POREN0001_0839), *P. somerae* DSM 23386 (PRJNA165425/A3GKDRAFT_10000), *P. pasteri* KA00676 (LSDJ0100000/HMPREF3184_00469), *P. buccalis* ATCC 35310 (PRJNA40669/HMPREF_R0056); Set II – (IMG Gene ID): *P. circumdentaria* DSM 103022 (2828460842), *P. crevioricanis* ATCC 55563 (2587859826), *P. macacae* DSM 20710 (2517121994), *P. gingivalis* ATCC 33277 (2843222782), *P. gulae* DSM 15663 (2519001822), *P. levii* DSM 23370 (2558308191), *P. gingivicanis* JCM 15907 (2587113678), *P. catoniae* ATCC 51270 (2559434490), *P. pasteri* KA00683 (2860175207), *P. uenonis* DSM 23387 (2528767153), *Porphyromonadaceae* sp. type 1 HMP subject159005010-visit2-bin3300007272 4 (Ga0104871_10051020);Set III – (NCBI nucleotide accession): *P. pasteri* JCM 30531 (LC014934.1), *Porphyromonadaceae* sp. type 1 C941 (JF803519.1), *Porphyromonadaceae* sp. type 1 DNF00129 (KU726636.1).
