# Supplementary material for: Protease activities of vaginal Porphyromonas species disrupt coagulation and extracellular matrix in the cervicovaginal niche
Source: NPJ Biofilms Microbiomes. 2022 Feb 21;8:8. doi: 10.1038/s41522-022-00270-7 (PMC8861167; doi:10.1038/s41522-022-00270-7)
Supplement: Supplementary file 1 — Supplemental Text & Figures [file 41522_2022_270_MOESM1_ESM.pdf]

Supplementary Information

Supplementary Note

Vaginal *Porphyromonas* species do not encode gingipain orthologs

Given the gingipain-like proteolytic activity observed in *P. asaccharolytica* and *P. uenonis* (Figures 1–2), we sought to confirm an earlier report that these organisms do not encode gingipain orthologs[1]. Using BLASTP, gingipain protein sequences (RgpA, RgpB, Kgp) were queried against *P. asaccharolytica* and *P. uenonis* genomes (Supplementary Figure 1A), yielding zero hits in *P. asaccharolytica* and two hits in each *P. uenonis* 23387 genome (IMG Genome ID 2585427891 and 2528311143); of note, these *P. uenonis* genomes were not queried in the previous analysis[1]. No BLASTP hits were identified in *P. uenonis* 60-3 as previously reported[1]. One hit resulted from the Kgp BLAST search only (Supplementary Table 3; L215DRAFT_00230/JCM13868DRAFT_00677; 27% sequence identity) and the other hit resulted from both the Kgp and RgpA BLAST queries (Supplementary Table 3; L215DRAFT_00128/JCM13868DRAFT_01423; 26–27% sequence identity). We further considered whether secreted proteases in *P. asaccharolytica* and *P. uenonis* share protein domains with gingipains. Evaluation of protein families (Pfams) in the gingipains revealed four conserved protein family (Pfam) domains, with RgpB containing one additional Pfam not found in RgpA or Kgp (Supplementary Table 4). All five Pfams were searched against all available genomes of *P. asaccharolytica* and *P. uenonis* to identify proteins containing gingipain Pfams (Supplementary Figure 1A). The peptidase C25 family (PF01364) search resulted in one hit in each of the genomes queried, while the cleaved adhesin domain (PF07675) returned hits in all *P. uenonis* strains, but none in *P. asaccharolytica* (Supplementary Table 4). To determine whether any hits from the BLAST search (Supplementary Table 3) and Pfam search (Supplementary Table 4) are gingipain orthologs, we performed a reciprocal BLASTP search against *P. gingivalis* (Supplementary Figure 1A). When the C25 peptidase-containing proteins identified in the Pfam search against *P. asaccharolytica* (Poras_0230) and *P. uenonis* (L215DRAFT_00971) were queried against *P. gingivalis* using BLASTP, PGN_0022 emerged as the only significant hit with 36–37% identity and 97% query coverage (Supplementary Table 5). Since PGN_0022 has been characterized as PorU, the type 9 secretion sortase enzyme in *P. gingivalis*[2], the C25 peptidase-containing proteins identified in *P. asaccharolytica* and *P. uenonis* are likely to function as PorU orthologs rather than gingipains. Querying the cleaved adhesin domain-containing proteins from *P. uenonis* against *P. gingivalis* revealed two uncharacterized proteins: PGN_1611 and PGN_1733 as the top BLASTP hits (Supplementary Table 5). The Lys-gingipain (Kgp) was identified as the fourth hit from both *P. uenonis* cleaved adhesin domain-containing protein BLASTP searches (Supplementary Table 5), but the percent identity and coverage are restricted to the shared Pfam. Furthermore, results from multiple sequence alignments with the *P. gingivalis* gingipains showed ≤20% identity for all *P. asaccharolytica* and *P. uenonis* sequences. In summary, since all BLASTP alignments were less than 200 residues, full length alignments of query and subject sequences were ≤20%, our Pfam search did not identify *P. asaccharolytica* or *P. uenonis* proteins that appeared to be gingipains, and reciprocal BLASTP searches did not return *P. gingivalis* gingipains as top hits, we concluded that these vaginal *Porphyromonas* species do not encode gingipain orthologs.

Supplementary References

1 O'Flynn, C. *et al.* Comparative Genomics of the Genus *Porphyromonas* Identifies Adaptations for Heme Synthesis within the Prevalent Canine Oral Species *Porphyromonas cangingivalis*. *Genome Biol Evol* **7**, 3397-3413, doi:10.1093/gbe/evv220 (2015).

2 Gorasia, D. G. *et al.* *Porphyromonas gingivalis* Type IX Secretion Substrates Are Cleaved and Modified by a Sortase-Like Mechanism. *PLoS Pathog* **11**, e1005152, doi:10.1371/journal.ppat.1005152 (2015).

Supplementary Figures

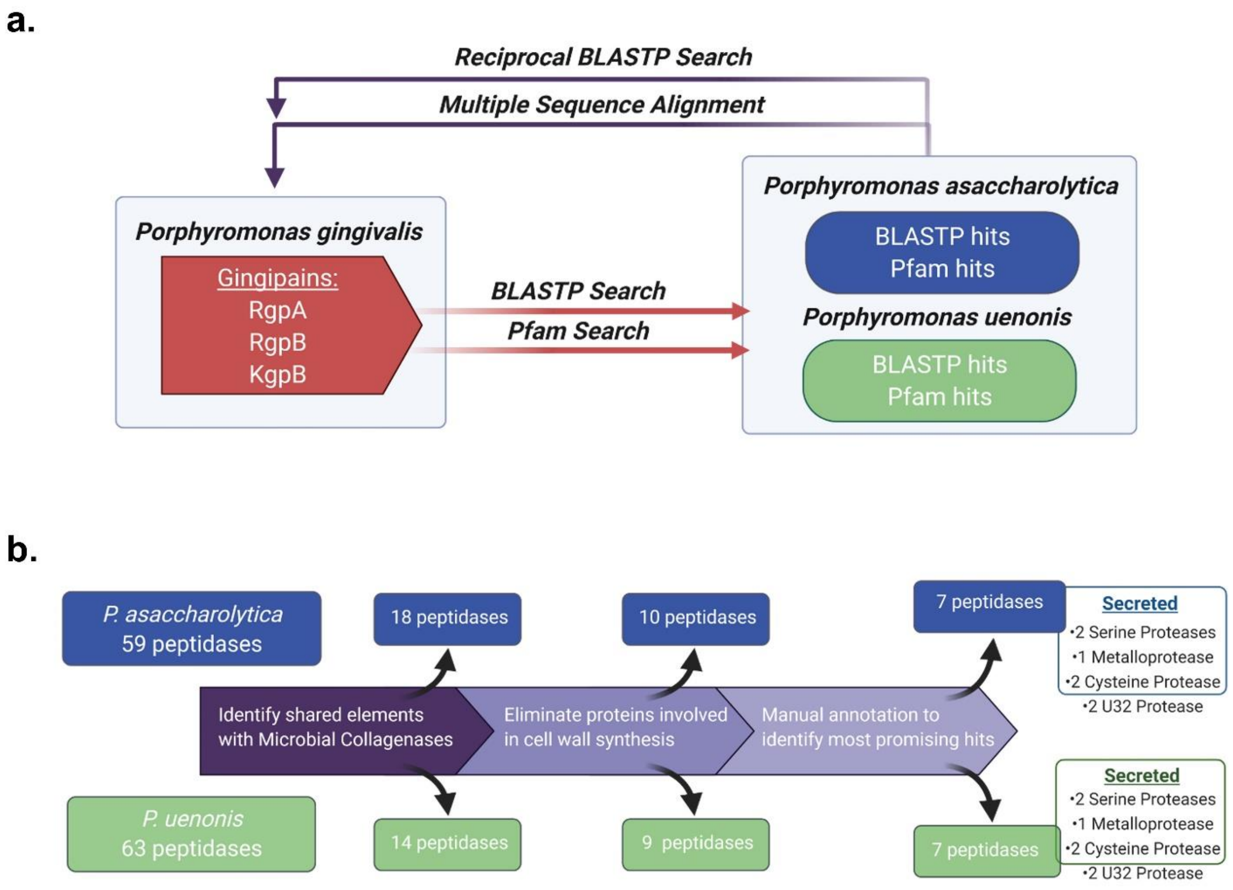

Supplementary Figure 1. Schematic of bioinformatics approaches. (a) Gingipain amino acid sequences for RgpA, RgpB and KgpB were used in BLASTP and Pfam searches against *P. asaccharolytica* and *P. uenonis* to search for gingipain orthologs. Resulting hits were subject to a reciprocal BLASTP search against *P. gingivalis* and a multiple sequence alignment with each gingipain. **(b)** Identification of candidate collagenases in *P. asaccharolytica* and *P. uenonis.* The MEROPS database was searched for all peptidases in *P. asaccharolytica and P. uenonis. Porphyromonas* peptidases were cross-referenced against protein annotation identifiers found in known and predicted microbial collagenases (BRENDA enzyme number: EC3.4.24.3). Candidate collagenases were explored in InterPro and UniProt to eliminate proteins involved in cell wall synthesis and identify the most promising hits. Images were prepared using BioRender.com**.**

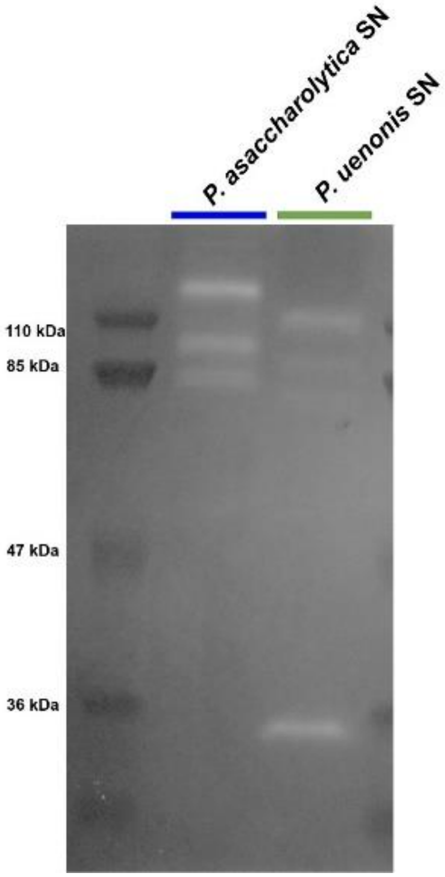

Supplementary Figure 2. Gelatin zymography of *P. asaccharolytica* and *P. uenonis* cell-free supernantants. Non-denaturing SDS-PAGE gelatin (type I collagen) zymogram loaded with 40 µg per well of *P. asaccharolytica* or *P. uenonis* supernatant. Zones of clearing were observed in Coomassie-stained gels after gel renaturing and enzyme activation. Representative zymogram from three independent experiments.

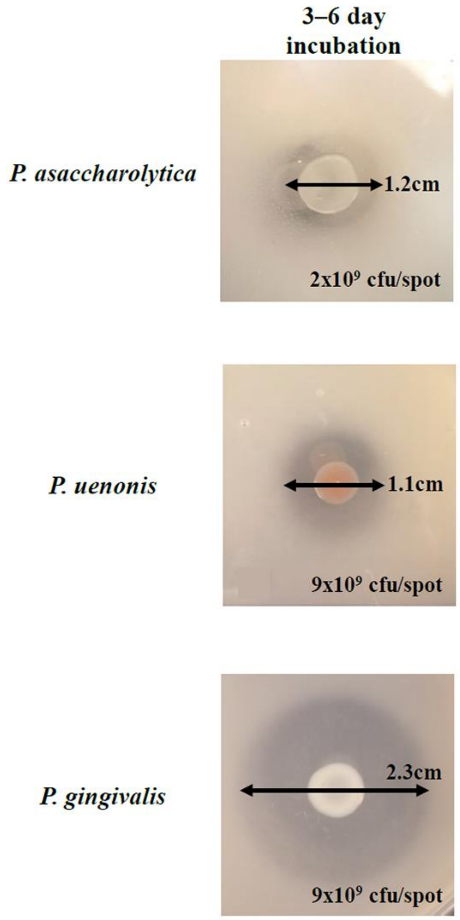

Supplementary Figure 3. Casein degradation ability of *P. asaccharolytica, P. uenonis* and *P. gingivalis.* Bacterial cell suspensions were spotted onto casein agar plates. Zones of clearing indicating casein degradation were measured after incubation for 3 or 6 days at designated doses of bacteria.

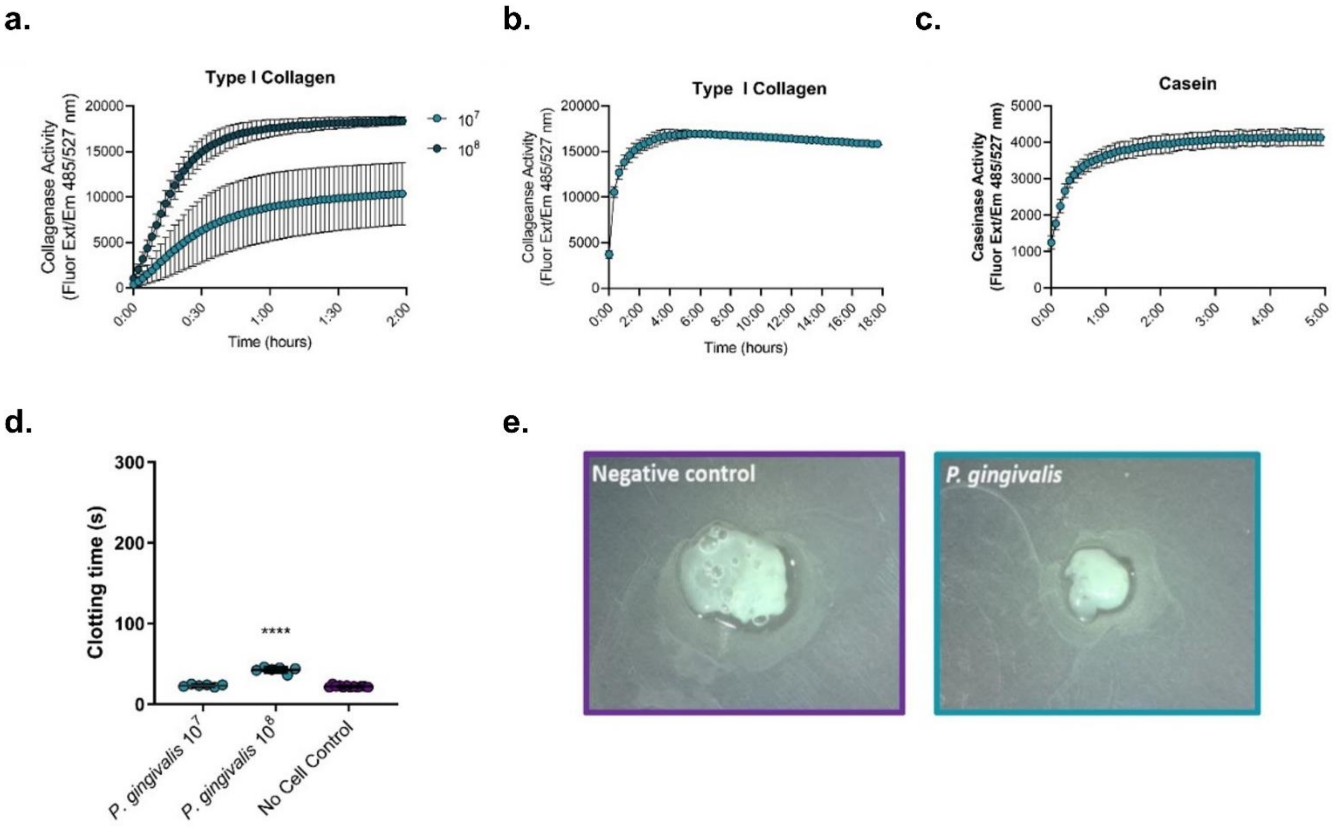

Supplementary Figure 4. Protease activity of *P. gingivalis.* (a) Cell suspensions of *P. gingivalis* at 10^7 or 10^8 CFU/reaction were incubated with fluorophore-conjugated type I collagen. Results are presented as mean ± standard error from two independent experiments performed in technical triplicate or quadruplicate. Collagen degradation was measured by detecting the increase in fluorescence over a two-hour time course. **(b)** Cell-free supernatants of *P. gingivalis* were incubated with fluorophore-conjugated type I collagen over an 18-hour time course. Results are presented as mean ± standard error from two independent experiments performed in technical triplicate. **(c)** Cell-free supernatants of *P. gingivalis* were incubated with fluorophore-conjugated casein over a five-hour time course; fluorescence was measured every ten minutes. Results are presented as mean ± standard error from two independent experiments performed in technical triplicate. **(d)** Time to fibrin clot formation after thrombin addition following pre-incubation of citrated plasma with no cell controls or cell suspensions of *P. gingivalis* at 10^7 or 10^8 CFU/reaction. Experiments were performed in technical duplicate and results are presented as mean ± SEM from two independent experiments. **(e)** Endpoint qualitative evaluation of fibrin clots (>30 minutes) after clotting time assay from no cell control or *P. gingivalis* cell suspension (10^8 CFU/mL).

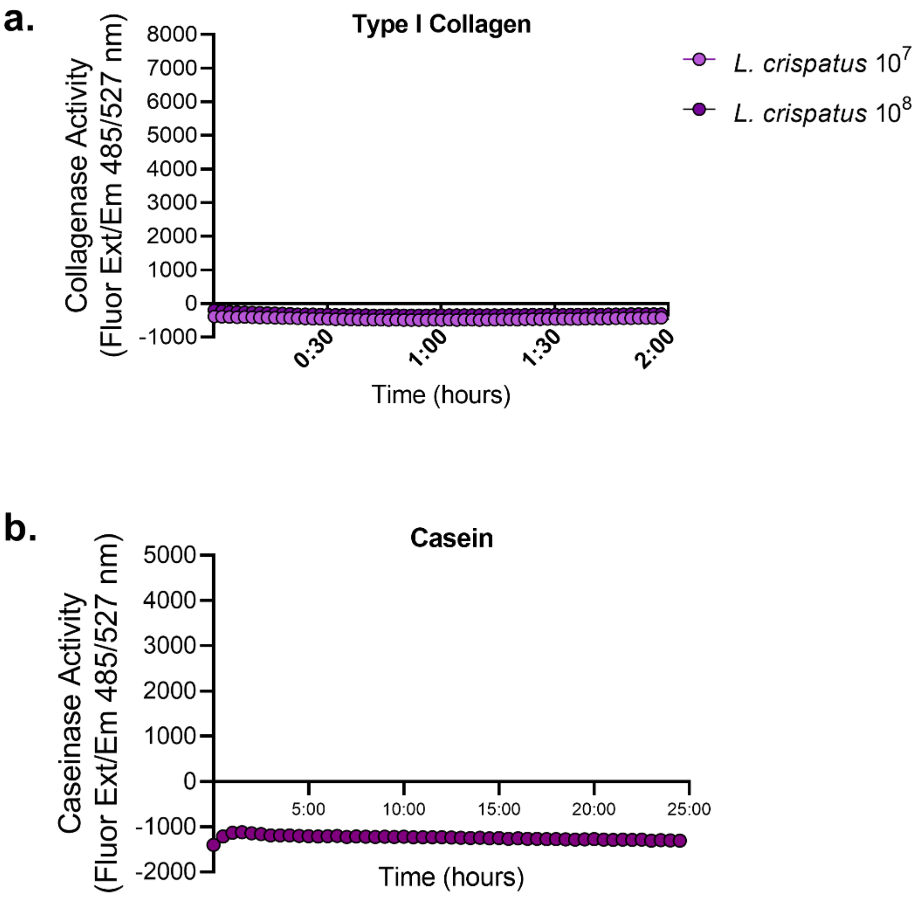

Supplementary Figure 5. *Lactobacillus crispatus* does not degrade type I collagen or casein. (a) Cell suspensions of *L. crispatus* at 10^7 or 10^8 CFU/reaction were incubated with fluorophore-conjugated type I collagen. Collagen degradation was measured by detecting the increase in fluorescence over a two-hour time course. Results are presented as mean ± standard deviation from one independent experiment performed in technical quadruplicate. **(b)** Cell suspensions of *L. crispatus* at $3x10^6$ CFU/reaction were incubated with fluorophore-conjugated casein. Casein degradation was measured by detecting the increase in fluorescence over a 24-hour time course. Results are presented as mean ± standard deviation from one independent experiment performed in technical triplicate.

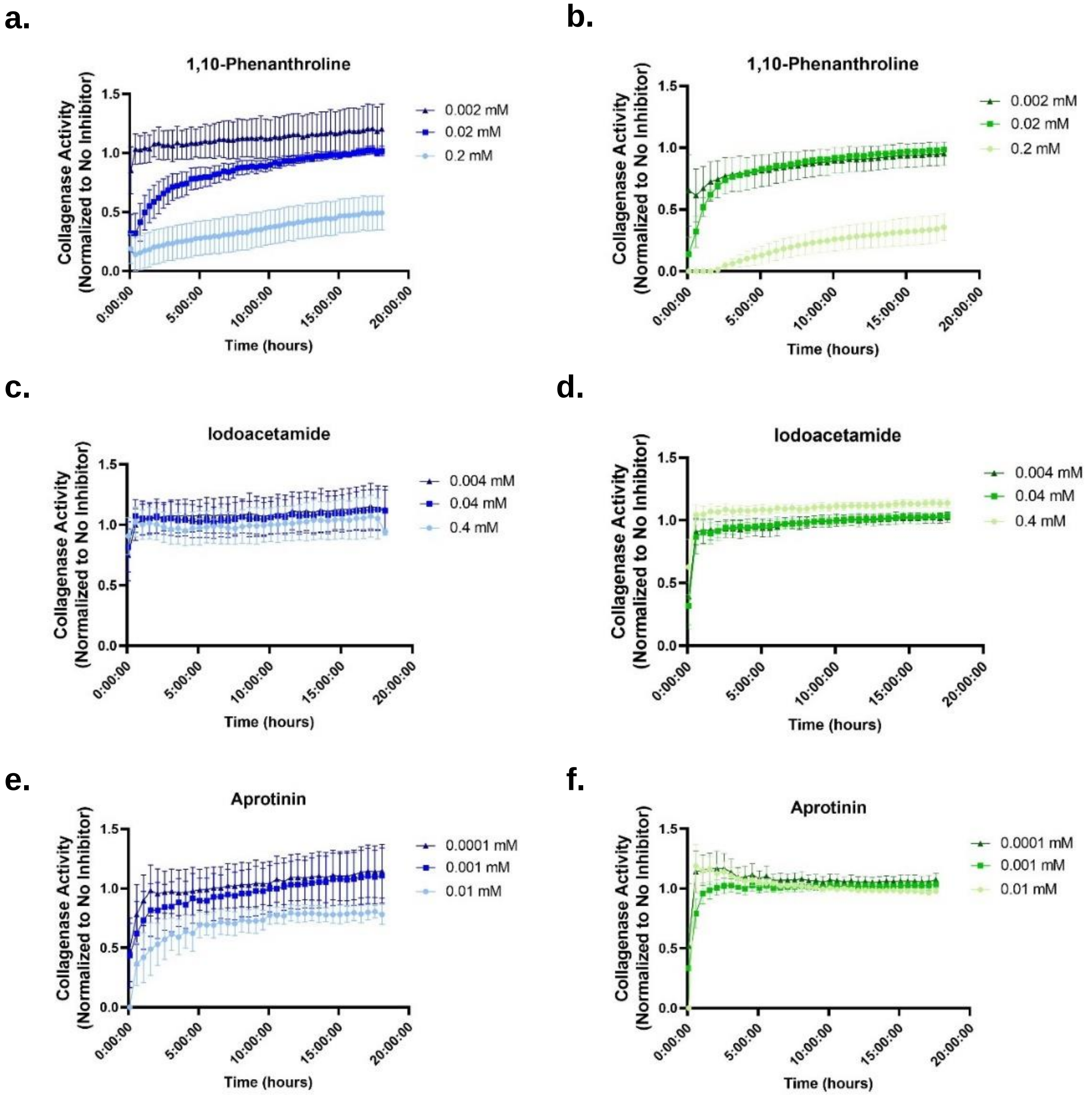
a.
1,10-Phenanthroline
Collagenase Activity (Normalized to No Inhibitor)
Time (hours)
0.002 mM
0.02 mM
0.2 mM
b.
1,10-Phenanthroline
Collagenase Activity (Normalized to No Inhibitor)
Time (hours)
0.002 mM
0.02 mM
0.2 mM
c.
Iodoacetamide
Collagenase Activity (Normalized to No Inhibitor)
Time (hours)
0.004 mM
0.04 mM
0.4 mM
d.
Iodoacetamide
Collagenase Activity (Normalized to No Inhibitor)
Time (hours)
0.004 mM
0.04 mM
0.4 mM
e.
Aprotinin
Collagenase Activity (Normalize to No Inhibitor)
Time (hours)
0.0001 mM
0.001 mM
0.01 mM
f.
Aprotinin
Collagenase Activity (Normalized to No Inhibitor)
Time (hours)
0.0001 mM
0.001 mM
0.01 mM

Supplementary Figure 6. Dose response inhibition of *P. asaccharolytica* and *P. uenonis* type I collagenase activity with protease inhibitors. Cell-free supernatants of **(a,c,e)** *P. asaccharolytica* (blue) or **(b,d,f)** *P. uenonis* (green) were incubated with fluorophore-conjugated type I collagen in the presence of three different doses of **(a–b)** the metalloprotease inhibitor 1,10-phenanthroline, **(c–d)** the cysteine protease inhibitor iodoacetamide, or **(e–f)** the serine protease inhibitor aprotinin. Collagen degradation was detected by measuring the increase in fluorescence over an 18-hour time course. Results are presented as a ratio normalized to the no inhibitor control and presented as mean ± standard error from four independent experiments or two independent experiments (aprotinin 0.01 mM dose) performed in technical triplicate.

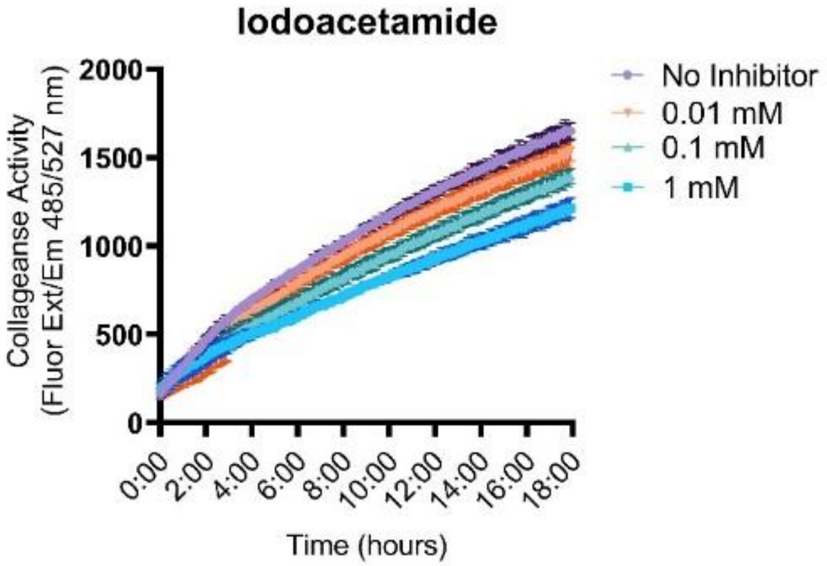

Supplementary Figure 7. Dose-dependent inhibition of *P. asaccharolytica* type I collagenase activity with the cysteine protease inhibitor iodoacetamide. *P. asaccharolytica* cell-free supernatant was incubated with fluorophore-conjugated type I collagen in the no inhibitor control or in presence of three different doses of the cysteine protease inhibitor iodoacetamide. Collagen degradation was measured by detecting the increase in fluorescence over an 18-hour time course. Results are presented as mean ± standard deviation from one independent experiment.

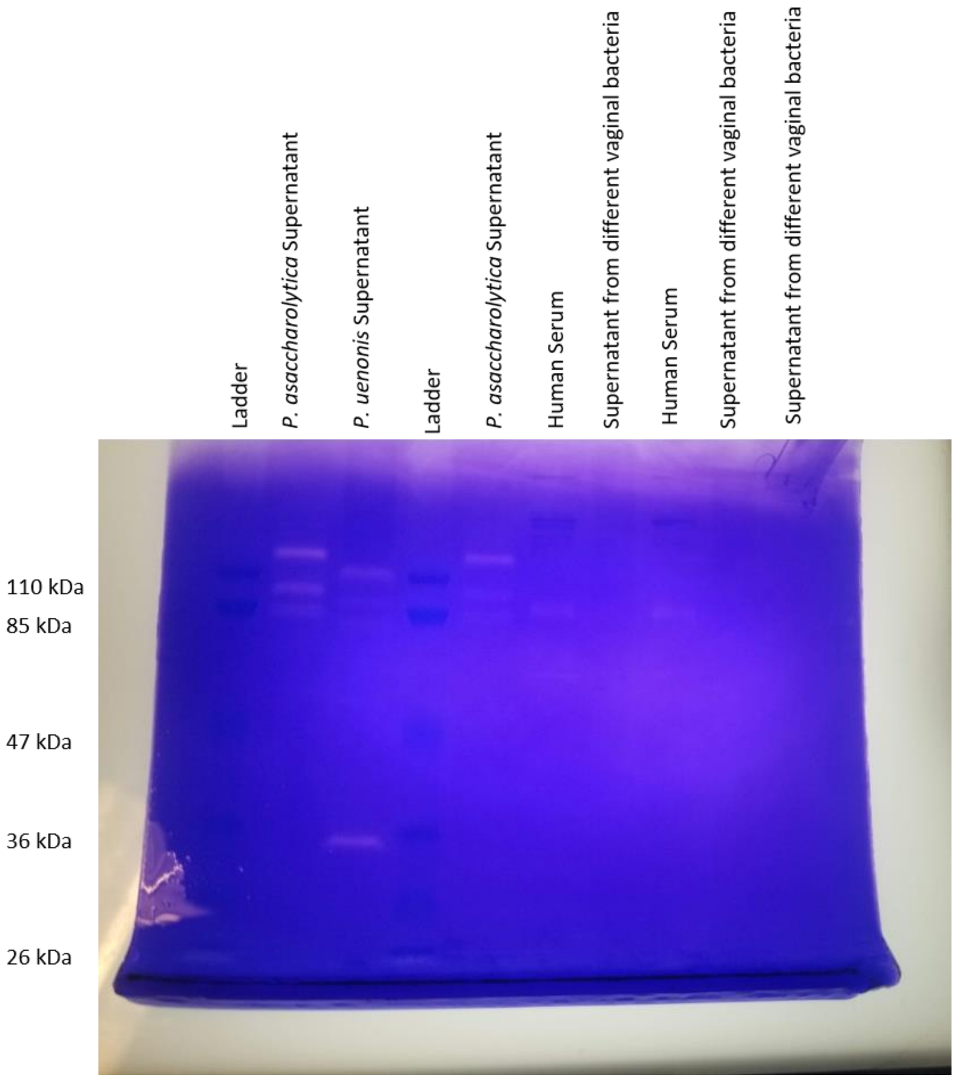

Supplementary Figure 8. Unprocessed, labelled non-denaturing SDS-PAGE gelatin (type I collagen) zymogram scan corresponding to the data presented in Supplementary Figure 2.

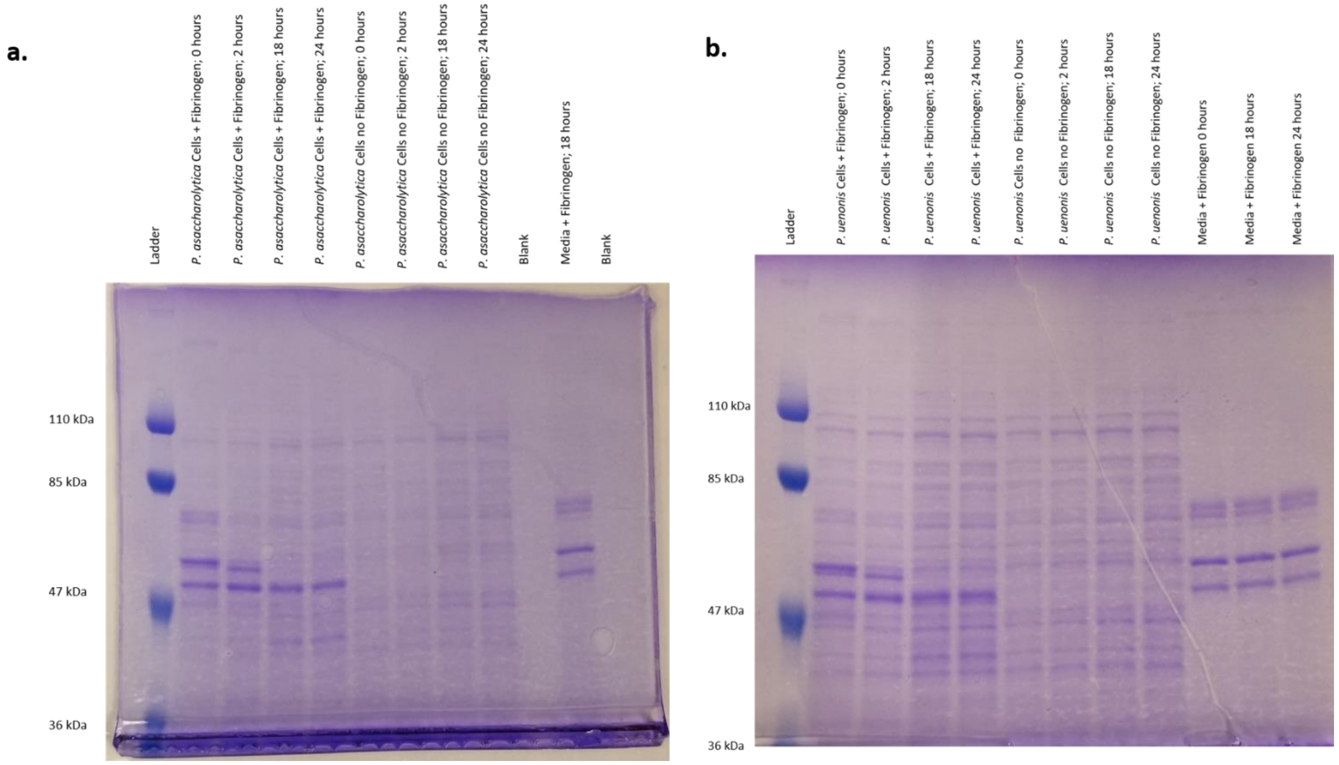

Supplementary Figure 9. Unprocessed, labelled SDS-PAGE gel scans corresponding to the data presented in **(a)** Figure 2a and **(b)** Figure 2b.

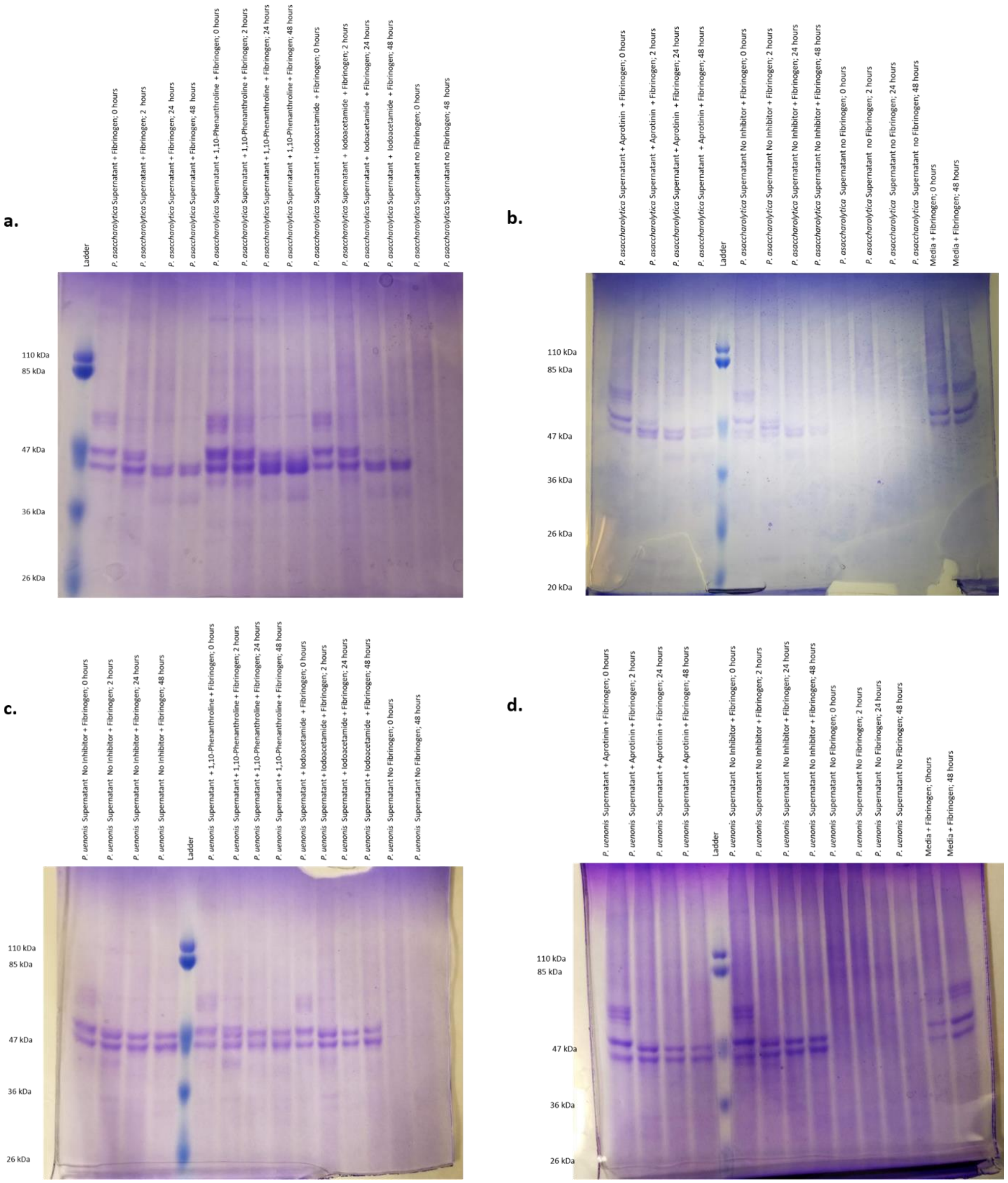

Supplementary Figure 10. Unprocessed, labelled SDS-PAGE gel scans corresponding to the data presented in (a,b) Figure 5a and (c,d) Figure 5b

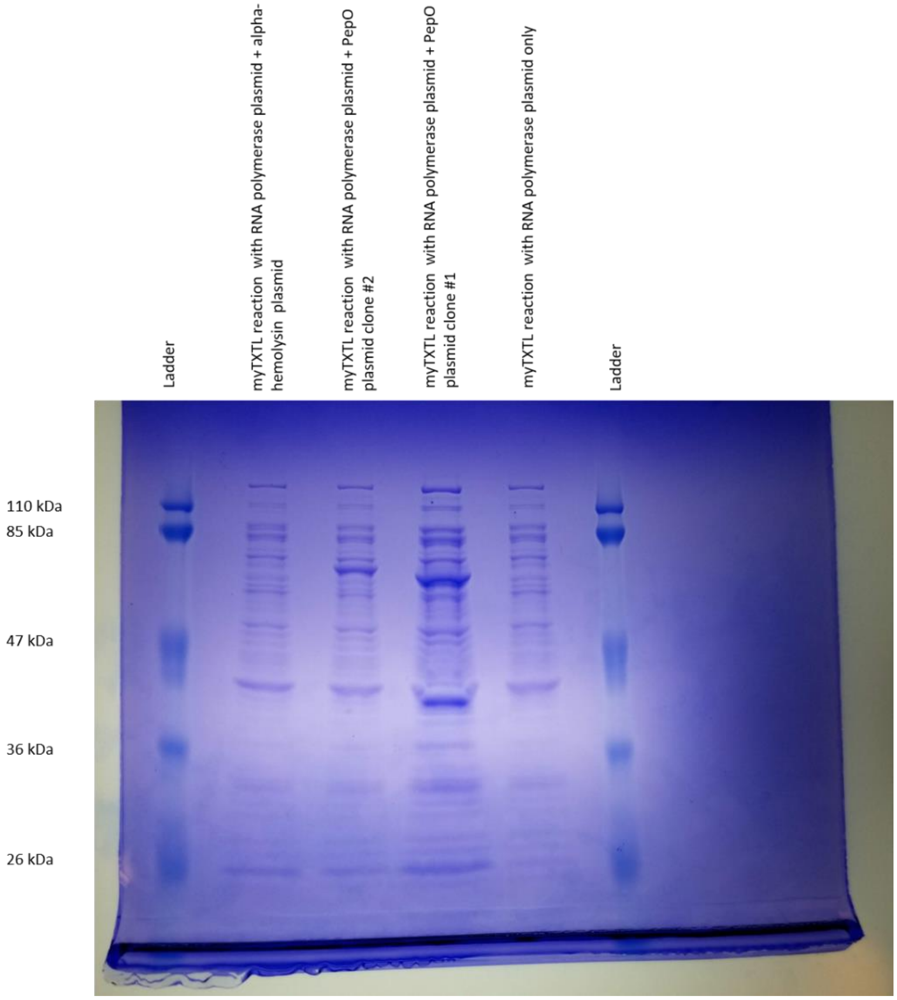

Supplementary Figure 11. Unprocessed, labelled SDS-PAGE gel scans corresponding to the data presented in Figure 7a.

Supplementary Tables

Supplementary Table 1. Type I collagenase activity of *Porphyromonas* species and *Lactobacillus crispatus* cell suspensions.

	10^7 CFU/reaction				10^8 CFU/reaction			
	Max RFU	Time to Max RFU	Slope	AUC*	Max RFU	Time to Max RFU	Slope	AUC*
P. asaccharolytica	2204.7 ± 1593.5	120 ± 0	1181 ± 119.1	2204 ± 207.7	5526 ± 628.2	120 ± 0	2857 ± 55.1	6170 ± 91.1
P. uenonis	2281 ± 168.7	120 ± 0	1111 ± 10.2	2349 ± 17.7	7389 ± 515.8	120 ± 0	3556 ± 62.7	9260 ± 86.74
P. gingivalis	10418	120 ± 0	4388 ± 439	15454 ± 746.3	18427	120 ± 0	6030 ± 316	30981± 184.3
L. crispatus	-392	0	-12	0	-201	0	-24	0

+Relative Fluorescence Units
*Area under the curve

Supplementary Table 2. Secreted collagenase and caseinase activity of *Porphyromonas* species.

	Type I Collagen				Casein			
	Max RFU	Time to Max RFU	Slope	AUC	Max RFU	Time to Max RFU	Slope	AUC
P. asaccharolytica	2396.8 ± 1542.4	1091 ± 1.7	99.8 ± 9.5	30991 ± 1742	3442 + 262.3	291 + 1.9	424 ± 24.8	13717 ± 270.7
P. uenonis	1059.5 ± 392.0	1093 ± 1.0	79.3 ± 2.8	15700 ± 552.4	3010.5 + 204.5	291.3 + 2.4	422.2 ± 21.9	11532 ± 209.3
P. gingivalis	17024 ± 373	335 ± 62.6	89 ± 11	292886 ± 413.9	4153 ± 236.6	265 ± 17.3	283 ± 22	78127 ± 202.8

+Relative Fluorescence Units
*Area under the curve

Supplementary Table 3. Gingipain BLASTP hits in *P. uenonis* DSM 23387.

Gingipain Query	BLASTP Hits[#]	E-value	% Sequence Identity	BLAST Hit Coverage (residues)	Subject Length
RgpA	L215DRAFT_00128* JCM13868DRAFT_01423⁺	1.00E-06	26%	308-502	1131
RgpB	N/A	N/A	N/A	N/A	N/A
Kgp	L215DRAFT_00128* JCM13868DRAFT_01423⁺	3.00E-06	26%	308-502	1131
	L215DRAFT_00230* JCM13868DRAFT_00677⁺	2.00E-06	27%	304-502	1131

*BLASTP hit in *P. uenonis* DSM23387 (IMG Genome ID 2528311143)

+BLASTP hit in *P. uenonis* DSM23387 (IMG Genome ID 2585427891)

[#]No BLASTP hits identified in *P. uenonis* 60-3

Supplementary Table 4. *P. asaccharolytica* and *P. uenonis* proteins containing gingipain Pfams.

		P. asaccharolytica		***P. uenonis***		
**Pfam ID[+]**	**Pfam Description**	CCUG 7834^	PR426713P-I	CCUG 48615^ (IMG Genome ID 2528311143)	CCUG 48615 (IMG Genome ID 2585427891)	60-3
PF01364	Peptidase family C25	2504824108[#]<br>Poras_0230	650259179[#]	2528766763[#]<br>L215DRAFT_00971	2587120245[#]	644453364[#]
PF03785	Peptidase family C25, C-terminal ig-like domain	N/A	N/A	N/A	N/A	N/A
PF08126	Propeptide_C25	N/A	N/A	N/A	N/A	N/A
PF07675	Cleaved Adhesin Domain	N/A	N/A	2528765925[#]<br>L215DRAFT_00128;<br>2528766027[#]<br>L215DRAFT_00230	257119003[#]	258711974[#]
PF10365	Domain of unknown function (DUF2436)	N/A	N/A	N/A	N/A	N/A
PF18630*	Peptidase M60 C-terminal domain	N/A	N/A	N/A	N/A	N/A

[+]Pfam IDs from *P. gingivalis* gingipains, RgpA, RgpB, Kgp

*Pfam only found in RgpB

[#]IMG/MER Gene IDs of pfam hit

^Type strains used in this study

Supplementary Table 5. Reciprocal BLASTP search of *P. asaccharolytica* and *P. uenonis* gingipain hits against *P. gingivalis*.

Strain	Query Gene ID/ Locus Tag	Top BLAST Hits in *P. gingivalis*	% Identity	Query Coverage	E-value	Gene Name	Function
P. asaccharolytica CCUG 7834	250482108; Poras_0230	PGN_0022	37%	97%	0	PorU	Type IX secretion system peptidase
P. uenonis CCUG 48615	2528766763; L215DRAFT_00971	PGN_0022	36%	97%	0	PorU	Type IX secretion system peptidase
	2528765925; L215DRAFT_00128	PGN_1611	28.89%	49%	9.00E-41	Leucine-Rich Repeat domain-containing protein	N/A
		PGN_0852	29.11%	34%	9.00E-36	Immunoreactive 47 kDa antigen	N/A
		PGN_1733	27.10%	17%	2.00E-09	Choice-of-anchor J domain-containing protein	N/A
		PGN_1728	26.63%	17%	7.00E-06	Kgp; Lys-gingipain	Gingipain
		PGN_1155	24.14%	12%	1.00E-04	Choice-of-anchor J domain-containing protein	N/A
	2528766027; L215DRAFT_00230	PGN_1733	25.37%	33%	1.00E-09	Choice-of-anchor J domain-containing protein	N/A
		PGN_1155	23.71%	37%	9.00E-07	Choice-of-anchor J domain-containing protein	N/A
		PGN_0335	28.92%	13%	2.00E-06	PKD domain-containing protein	N/A
		PGN_1728	24.77%	37%	1.00E-05	Kgp; Lys-gingipain	Gingipain

Supplementary Table 6. Candidate collagenases in *P. uenonis* 60-3 and corresponding genes in *P. uenonis* CCUG 48615.

Accession ID (*P. uenonis* 60-3)	*P. uenonis* CCUG 48615*	Percent Identity	Protein Name	Protease Type	All Feature IDs in *P. uenonis* 60-3	Differences in Collagenase Feature IDs^
PORUE0001_1484	L215DRAFT_00939	52%	Peptidase S1 domain-containing protein	Serine	IPR13783; IPR026444; TIGR0483	N/A
PORUE0001_0731	L215DRAFT_01109	72%	Trypsin	Serine	IPR13783; IPR026444; TIGR0483; SF49265	IPR036116 (Fn-3 domain); SF49265; (Fn3 type III)
PORUE0001_1366	L215DRAFT_01080	98%	Peptidase, S9A/B/C family, catalytic domain protein	Serine	SSF53474	N/A
PORUE0001_0281	L215DRAFT_00076	91%	Peptidase family M13	Metallo	IPR024079	N/A
PORUE0001_0568	L215DRAFT_00076	93%	Peptidase family M13	Metallo	IPR024079	N/A
PORUE0001_0451	L215DRAFT_00099	95%	Putative thiol protease/hemagglutinin	Cysteine	IPR026444; TIGR0483	N/A
PORUE0001_1363	L215DRAFT_01083	93%	Putative thiol protease/hemagglutinin	Cysteine	IPR026444; TIGR0483	N/A
PORUE0001_1169	L215DRAFT_01354	97%	Peptidase, S9A/B/C family, catalytic domain protein	Serine	IPR029058	N/A
PORUE0001_1130	L215DRAFT_01540	97%	Peptidase, U32 family	Unknown (U32)	PF01136; PF12392	N/A
PORUE0001_0329	L215DRAFT_00228	98.80%	Peptidase, U32 family	Unknown (U32)	PF01136	N/A

*Top BLASTP hit
^Present in *P. uenonis* 60-3 only

Supplementary Table 7. Intrastrain and interstrain identity of *Porphyromonas* candidate collagenases.

	P. asaccharolytica DSM 20707		*P. uenonis* DSM 23387		
**Protease Type**	Gene	Intrastrain Identity[1]	Gene[2]	Intrastrain Identity[1]	Interspecies Identity[1]
**Ig-containing Serine Protease**	Poras_1474	45.1%	Poru_01109	35.2%	77.5%
	Poras_0168		Poru_00939		55.6%
**M13 Metalloprotease**	Poras_0079	N/A	Poru_00076	N/A	92.7%
**C10 Protease**	Poras_1659	31.3%	Poru_01083	28.8%	67.2%
	Poras_0891		Poru_00099		72.8%
**U32 Collagenase**	Poras_0217	29.3%	Poru_01540	29.0%	90.6%
	Poras_0873		Poru_00228		97.3%

[1]Amino acid identities determined using Clustal Omega multiple sequence alignments

[2]Locus tags correspond to L215DRAFT_XXXXX
