# Supplementary material for: Protease activities of vaginal Porphyromonas species disrupt coagulation and extracellular matrix in the cervicovaginal niche
Source: NPJ Biofilms Microbiomes. 2022 Feb 21;8:8. doi: 10.1038/s41522-022-00270-7 (PMC8861167; doi:10.1038/s41522-022-00270-7)
Supplement: Supplementary file 2 — Reporting Summary [file 41522_2022_270_MOESM2_ESM.pdf]

nature portfolio

Corresponding author(s): Laura Sycuro

Last updated by author(s): Oct 14, 2021

Reporting Summary

Nature Portfolio wishes to improve the reproducibility of the work that we publish. This form provides structure for consistency and transparency in reporting. For further information on Nature Portfolio policies, see our Editorial Policies and the Editorial Policy Checklist.

Statistics

For all statistical analyses, confirm that the following items are present in the figure legend, table legend, main text, or Methods section.

n/a	Confirmed	
☐	☒	The exact sample size (*n*) for each experimental group/condition, given as a discrete number and unit of measurement
☐	☒	A statement on whether measurements were taken from distinct samples or whether the same sample was measured repeatedly
☐	☒	The statistical test(s) used AND whether they are one- or two-sided<br>*Only common tests should be described solely by name; describe more complex techniques in the Methods section.*
☒	☐	A description of all covariates tested
☐	☒	A description of any assumptions or corrections, such as tests of normality and adjustment for multiple comparisons
☐	☒	A full description of the statistical parameters including central tendency (e.g. means) or other basic estimates (e.g. regression coefficient) AND variation (e.g. standard deviation) or associated estimates of uncertainty (e.g. confidence intervals)
☐	☒	For null hypothesis testing, the test statistic (e.g. *F*, *t*, *r*) with confidence intervals, effect sizes, degrees of freedom and *P* value noted<br>*Give P values as exact values whenever suitable.*
☒	☐	For Bayesian analysis, information on the choice of priors and Markov chain Monte Carlo settings
☒	☐	For hierarchical and complex designs, identification of the appropriate level for tests and full reporting of outcomes
☒	☐	Estimates of effect sizes (e.g. Cohen's *d*, Pearson's *r*), indicating how they were calculated

Our web collection on statistics for biologists contains articles on many of the points above.

Software and code

Policy information about availability of computer code

Data collection	We disclose that no unpublished software was used. Data was downloaded from the MEROPs database (https://www.ebi.ac.uk/merops/) and uniprot database (https://www.uniprot.org/) as described in the methods and supplementary methods sections. For fluorescence-based protease assays and quantitative clotting assays, data was collected using BioTek Gen5 software (https://www.biotek.com/products/software-robotics-software/gen5-microplate-reader-and-imager-software/).
Data analysis	We disclose that no unpublished software was used. Data were analyzed using GraphPad Prism Version 8 Software (https://www.graphpad.com/) and Stata Version 15 Software (https://www.stata.com/) as described in the methods section.

For manuscripts utilizing custom algorithms or software that are central to the research but not yet described in published literature, software must be made available to editors and reviewers. We strongly encourage code deposition in a community repository (e.g. GitHub). See the Nature Portfolio guidelines for submitting code & software for further information.

Data

Policy information about availability of data

All manuscripts must include a data availability statement. This statement should provide the following information, where applicable:

- Accession codes, unique identifiers, or web links for publicly available datasets
- A description of any restrictions on data availability
- For clinical datasets or third party data, please ensure that the statement adheres to our policy

The authors confirm that the data supporting the findings of this study are available within the paper and Supplementary files. Additional data are available from the corresponding author upon request.

Field-specific reporting

Please select the one below that is the best fit for your research. If you are not sure, read the appropriate sections before making your selection.

☒ Life sciences ☐ Behavioural & social sciences ☐ Ecological, evolutionary & environmental sciences

For a reference copy of the document with all sections, see nature.com/documents/nr-reporting-summary-flat.pdf

Life sciences study design

All studies must disclose on these points even when the disclosure is negative.

Sample size	No method was used to calculate the sample size. The sample size was equal or greater than 2 in all cases and sample size was determined based on the number of replicates needed to minimize variation between experiments.
Data exclusions	No data was excluded from this work
Replication	Our data shows experiments conducted with two or more independent experiments run with technical replicates (duplicate, triplicate or quadruplicate, depending on the assay) as indicated in figure legends. Replicates were successful with one exception: in P. asaccharolytica supernatant assays with the cysteine inhibitor, iodoacetamide, we observed that select independent experiments showed a dose-dependent trend for inhibition of proteolytic activity (shown in Supplementary Figure 7). However, this trend was not repeated in all experiments and was not present when independent experiments were combined. Therefore, we reported this trend in the supplementary figures and results section, but showed the averaged data across all experiments in our main figure (Fig 4a,b iodoacetamide treatments).
Randomization	No randomization was used. Bacterial strain cell suspensions and supernatants were compared to negative controls (media only) and exposed to different treatments (ex: protease inhibitors).
Blinding	Blinding was used during the qualitative components of the clotting assays. Researchers were blinded to the sample types while assessing the time to formation of clots.

Reporting for specific materials, systems and methods

We require information from authors about some types of materials, experimental systems and methods used in many studies. Here, indicate whether each material, system or method listed is relevant to your study. If you are not sure if a list item applies to your research, read the appropriate section before selecting a response.

Materials & experimental systems

n/a	Involved in the study	
☒	☐	Antibodies
☒	☐	Eukaryotic cell lines
☒	☐	Palaeontology and archaeology
☒	☐	Animals and other organisms
☒	☐	Human research participants
☒	☐	Clinical data
☒	☐	Dual use research of concern

Methods

n/a	Involved in the study	
☒	☐	ChIP-seq
☒	☐	Flow cytometry
☒	☐	MRI-based neuroimaging
